# Molecular Signatures in *Arabidopsis thaliana* in Response to Insect Attack and Bacterial Infection

**DOI:** 10.1371/journal.pone.0058987

**Published:** 2013-03-25

**Authors:** Pankaj Barah, Per Winge, Anna Kusnierczyk, Diem Hong Tran, Atle M. Bones

**Affiliations:** Department of Biology, Norwegian University of Science and Technology (NTNU), Trondheim, Norway; Virginia Tech, United States of America

## Abstract

**Background:**

Under the threat of global climatic change and food shortages, it is essential to take the initiative to obtain a comprehensive understanding of common and specific defence mechanisms existing in plant systems for protection against different types of biotic invaders. We have implemented an integrated approach to analyse the overall transcriptomic reprogramming and systems-level defence responses in the model plant species *Arabidopsis thaliana* (*A. thaliana* henceforth) during insect *Brevicoryne brassicae* (*B. brassicae* henceforth) and bacterial *Pseudomonas syringae pv. tomato strain DC3000* (*P. syringae* henceforth) attacks. The main aim of this study was to identify the attacker-specific and general defence response signatures in *A. thaliana* when attacked by phloem-feeding aphids or pathogenic bacteria.

**Results:**

The obtained annotated networks of differentially expressed transcripts indicated that members of transcription factor families, such as *WRKY, MYB, ERF, BHLH and bZIP,* could be crucial for stress-specific defence regulation in *Arabidopsis* during aphid and *P. syringae* attack. The defence response pathways, signalling pathways and metabolic processes associated with aphid attack and *P. syringae* infection partially overlapped. Components of several important biosynthesis and signalling pathways, such as salicylic acid (SA), jasmonic acid (JA), ethylene (ET) and glucosinolates, were differentially affected during the two the treatments. Several stress-regulated transcription factors were known to be associated with stress-inducible microRNAs. The differentially regulated gene sets included many signature transcription factors, and our co-expression analysis showed that they were also strongly co-expressed during 69 other biotic stress experiments.

**Conclusions:**

Defence responses and functional networks that were unique and specific to aphid or *P. syringae* stresses were identified. Furthermore, our analysis revealed a probable link between biotic stress and microRNAs in *Arabidopsis* and, thus gives indicates a new direction for conducting large-scale targeted experiments to explore the detailed regulatory links between them. The presented results provide a comparative understanding of *Arabidopsis* – *B. brassicae* and *Arabidopsis* – *P. syringae* interactions at the transcriptomic level.

## Introduction

Plants are sessile organisms that are unable to escape biotic and abiotic stresses. As a result, they have evolved flexibility in their responses to changing environmental conditions, such as light, drought, temperature, the available nutritional supply and biotic invasion. Different types of biotic invasions, such as insect, bacterial, fungal and viral invasions, represent a severe threat to agricultural production worldwide [1]. Some responses of host plants to different stress conditions are very general and provide protection from a variety of invading organisms, whereas others are more specific and target particular types of attackers. Highly complex and often connected signalling pathways, regulating numerous metabolic networks, coordinate plant responses to different stress conditions. Over the last decade or so, clear advances have been made in understanding how defence responses are orchestrated in higher plants. The development of microarray technology has allowed monitoring of expressional changes in thousands of genes simultaneously, and this technology has now become a major tool for examining plant stress biology. Most of these studies have adopted *A. thaliana* as a model plant organism because of the vast amount of genomic information made available for this species with the completion of the *A. thaliana* genome sequence and advanced annotation of *A. thaliana* genes [2]. Analysing the regulation of gene expression under various stress conditions has revealed that the early defence responses of a plant to different stress factors often overlap and engage the same sets of genes [3]. It has also become evident that different types of plant invaders may induce substantially different changes in the host plant transcriptome. Furthermore, studies on plants subjected to various treatments indicate that the induced defences can be both general – being commonly manifested regardless of the type of applied treatment; and specific – providing protection from a certain type of stress [4], [5]. In many cases, however, the multidimensional level of network crosstalk makes it challenging to recognise which of the observed responses are general and which are more stress specific [6], [7].

Aphids are one of the world’s major insect pests, causing serious economic damage to a range of temperate and tropical crops [8]. Aphids use their mouthparts, formed into a stylet-like structure, to pierce plant tissue in the search for sieve elements (SEs) containing their primary food source: phloem sap [9], [10]. Feeding by an aphid causes minimal wounding, as its stylet proceeds mostly intercellulary and is inserted only into selected cells on its way to the phloem tissue [11]. However, the disruption of cell walls and membranes of the pierced cells is likely to be the first factor triggering a plant response. In addition, the salivary secretions lubricating the stylet throughout its pathway through plants tissues and injected into SEs during feeding contain molecular signatures that activate plant defences. Therefore, despite their stealthy feeding, aphids are strong inducers of plant defences against them. Recently Kuśnierczyk *et al.* reported the timing and dynamics of early *Arabidopsis* defence responses [12] to an aphid attack.

*P. syringae* is a bacterial leaf pathogen that causes extensive chlorosis and necrotic spots [13]. Many strains of *P. syringae* are pathogenic in the model plant *A. thaliana*, and *P. syringae* is therefore widely used to study plant – pathogen interactions under laboratory conditions. *P. syringae* enters host tissues through wounds or natural openings such as stomata, and in susceptible plants, it multiplies to high concentrations in intercellular spaces [14]. The ability of *P. syringae* to multiply endophytically is dependent on its type III secretion pathway enabling the secretion of proteins into the apoplast. These proteins interact with the cell wall and plasma membrane and are directly translocated into the cytoplasm of host cells [15]. Several strains of *P. syringae* produce coronatine, a molecule that mimics endogenous plant jasmonyl-L-isoleucine and an activator of the jasmonic acid signalling pathway [16]. By doing so, the bacteria manipulate host responses, suppressing salicylic acid defences through the activation of jasmonic acid signalling [17], [18].

A great number of experiments conducted to assess plant responses to different stresses have made substantial contributions to our understanding of the induced defences of plants. However, the comparison of independent experiments and extraction of meaningful information from such comparisons is complicated and difficult in most cases, mainly due to the lack of common standards regarding how to grow plants, conduct expression profile experiments, and finally, how to evaluate the resulting gene expression data [19]. In recent years, integrated approaches, such as systems biology methods, have been evolving, providing promising tools for studying plant stress responses [20], [21]. Scientists intend to go beyond simple functional enrichment analyses to understand the molecular basis of genome-scale microarray experiments. Methods inspired by systems biology utilise lists of differentially expressed genes ranked by biological criteria to search for the distribution of blocks of functionally related genes without imposing any artificial threshold. Such ranked lists of genes can be arranged into functional classes, pathways and biological processes. Co-expression or co-regulation of particular genes can indicate their involvement in similar biological processes, meaning that individual modules of genes can be attributed to specific biological processes. Using this basic concept, modular network topology-based analysis has been proven to be useful in identifying functional modules of genes [22]. In a recent co-expression study, Weston and co-workers showed how a co-expression network-based analysis can be used for understanding population-level adaptive physiological responses of plants to abiotic stress [23].

MicroRNAs (microRNAs) are small, non-coding RNAs that play critical roles in post-transcriptional gene regulation and stress-inducible transcriptional regulation in *Arabidopsis* [24]. In plants, mature microRNAs pair with complementary sites on mRNAs, subsequently leading to the cleavage and degradation of the mRNAs. Many microRNAs target mRNAs that encode transcription factors and, thus, influence the expression of many genes whose regulation is controlled by these transcription factors [25]. The identification, detection, regulation and functional analysis of microRNAs associated with biotic stress remains a great challenge. In contrast, information about plant stress-responsive genes and their transcription factor binding sites is available to some extent in several databases [26], [27], [28], [29], [30]. Integration of such publicly available knowledge bases with experimental approaches would provide useful insights in understanding the plant defence responses to different biotic stresses.

In this manuscript, we present such an integrated approach to explore the common (general) and attacker-specific defence responses of *A. thaliana* subjected to two different types of biotic invaders: phloem-feeding aphids (*B. brassicae*) and pathogenic bacteria (*P. syringae*). To allow comparison between the obtained gene expression profiles and the observed regulation of gene pathways involved in defence against the aphid and the bacterium, the same growth and experimental conditions were used in the two simultaneous experimental setups. Transcriptional changes resulting either from infestation with *B. brassicae* or infection with *P. syringae* were assessed with the use of full-genome Arabidopsis microarrays (the data have been deposited in GEO with accession numbers GSE39245 and GSE39246).

Two sets of differentially expressed genes, corresponding to the plant responses to either aphid or bacterial treatment, were created as the outcome of the microarray data analysis. In an attempt to integrate the resulting data with publicly available knowledge extracted from several different databases as well as from published results of other experiments, these two differentially regulated gene sets were subsequently analysed through a set of computational approaches. The following analyses were incorporated into the presented work: an analysis of enriched functional categories or processes; exploration of potential connections between microRNAs and biotic stress-inducible transcriptional regulation during insect and bacterial attack; cross-validation of the aphid- and *Pseudomonas*-regulated genes using a co-expression network constructed from a compendium of 69 other biotic stress microarray datasets complied in the CORNET tool [31] (https://cornet.psb.ugent.be/).

## Results and Discussion

### Overall Changes in the *Arabidopsis* Transcriptome in Response to Insect and Bacterial Attack

To explore the complexity of the transcriptional changes induced by the different examined *A. thaliana* attackers, we compared the overlap between the obtained gene sets. From the results, it is evident that the transcriptional responses of *A. thaliana* to these very different attackers are massive. Aphid infestation and *P. syringae* infection resulted in significant differential regulation of 4,979 (2,803 up-regulated, 2,176 down-regulated) and 3,199 (1,634 up, 1,565 down) genes, respectively **(** **Table 1** and **Tables S4, S5)**. Although aphids and bacteria exhibit very different modes of action and trigger a highly dissimilar signal signature, a large number of *Arabidopsis* genes were expressed in response to both attackers. There were 1,597 common genes affected after both aphid infestation and *P. syringae* treatment. A total of 3,382 genes (1,963 up, 1,419 down) showed aphid-specific expression, while 1602 genes (842 up, 760 down) showed *P. syringae*-specific expression (**Table S6**). In the common set of genes, there were a total of 186 genes that showed opposite expression patterns in the two experiments. Of these genes, 117 were up-regulated under aphid and down-regulated under *P. syringae* attack, while 69 genes were down-regulated under aphid and up-regulated under *P. syringae* attack. Out of the 117 genes that were up-regulated in the aphid and down-regulated in the *P. syringae* experiment, 17 have been reported to be transcription factors. Six of these transcription factors are members of the *ERF/AP2* transcription factor family. Among them *ERF10*4, which is regulated by *MPK6*, is a key controller of innate immunity and dehydration stress [32].

**Table 1:** Overall summary of the differentially regulated genes in *A. thaliana* during *Brevicoryne brassicae* (aphid) attack or *P. syringae* (bacteria) infection.

Category	No. of Genes	Up- regulated	Down- regulated	No. of TF
Differentially expressed during Aphid exp.	4979	2803	2176	303
Differentially expressed during *P. syringae* exp.	3199	1634	1565	191
*Common to both exp.	1597	723	688	87
Only Aphid	3382	1963	1419	216
Only *Pseudomonas*	1602	842	760	104

* In the common set of genes, 186 genes showed opposite expression patterns during the two experiments. Among these genes, 117 were up-regulated under aphid and down-regulated under *P. syringae* attack, while 69 genes were down-regulated under aphid and up-regulated under *P. syringae* attack.

In total, 303 transcription factors were found to be affected by the aphid treatment, while 191 transcription factors showed altered expression under *P. syringae* infection. The common category (differentially expressed during both aphid infestation and *P. syringae* treatment) included 87 known Arabidopsis transcription factors. The analysis also identified 216 transcription factors that were differentially regulated only during the aphid treatment and 104 transcription factors that were differentially regulated only during *P. syringae* infection. The annotated network of these transcripts showed that some of the differentially expressed transcription factors could be crucial for stress-specific defence responses in *A. thaliana* plants.

Analysis of overrepresented gene ontologies (GO) in *A. thaliana* indicates rigorous reprogramming of several biological processes. As seen from the **Table 1**, a large number of genes were differentially regulated in *A. thaliana* during both the aphid and *P. syringae* experiments, which indicated that intense transcriptional reprogramming took place. A network-based analysis of the corresponding GO terms under the *Biological Process* classification using ClueGO (correction method = Bonferroni, kappa score ≥0.3) in the common aphid-specific and *P. syringae*-specific transcript dataset was performed.

**Figure 1:**
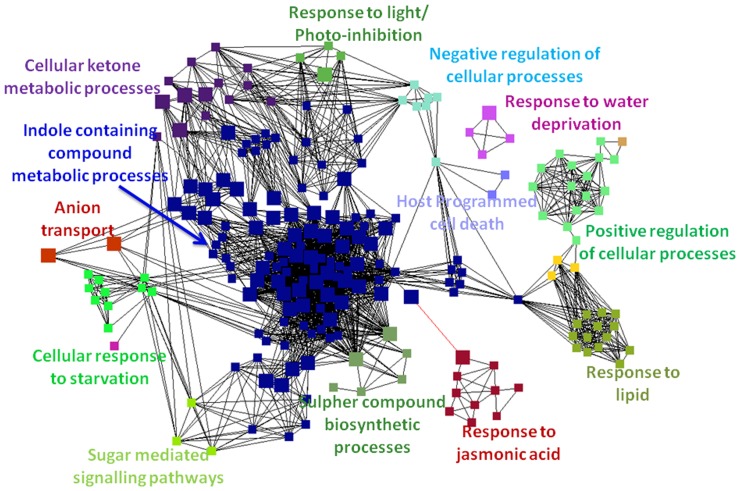
Over-represented GO-categories in the common gene list. Network representations of enriched GO categories among the genes that were differentially regulated during both experiments. Figure generated from the functionally grouped networks of enriched GO categories among genes whose expression is induced by both the aphid and pathogenic bacterium treatments. GO terms are represented as nodes based on their kappa score (≥0.3); only networks with at least three nodes are represented. The node size indicates the significance of the term’s enrichment. The edges are related to the relationships between the selected terms, which are defined based on the genes that are shared in a similar way. The label of the most significant term is used as the leading group term. Visualisation was conducted using Cytoscape 2.7.0.

When this analysis was applied to the list of 1,597 common genes whose expression was affected during both of the experiments, 17 significantly overrepresented categories were identified (some of these categories are shown in **Figure 1**.) Most of the cellular and metabolic processes were clustered in distinctly separate modules, and there were few highly interconnecting overrepresented processes. More than half of the genes from the common list were involved in central metabolic and cellular processes, such as electron transport and energy pathways located in the plastid. Some of the most significant categories were indole-containing compound metabolic processes, host localised cell death, cellular responses to starvation, downregulation of photosynthesis, responses to jasmonic acid, sulphur compound biosynthetic processes, and negative regulation of cellular processes. Analysis of the modules showed that the majority of the jasmonic acid responsive genes were up-regulated by both treatments, but the number of genes and their degree of induction were markedly higher in the *P. syringae-*treated plants, which may be due to the effects of coronatine (**file S11**). It has been previously reported that *P. syringae* uses the virulence factor coronatine (*COR*) as a mimic of jasmonyl-l-isoleucine (*JA-Ile*) [16], [33]. The coronatin-regulated *A. thaliana* genes reported in Thilmony R. et al., 2006 [34] show strong overlap with our *P. syringae* data. More than 450 genes reported as coronatin-regulated by Thilmony R. et al. show highly similar expression patterns in the two datasets (data not shown).

Tryptophan-derived indolic compounds, such as indolic glucosinolates (*iGS*) and indolic-derived phytoalexins, are an important component elicitor-induced responses in Arabidopsis plants [35], [36]. The biosynthesis of tryptophan-derived indolic compounds was up-regulated under both treatments but was stronger induced by aphid infestation (**file S4** and **S5**). Two of the affected modules, cellular responses to starvation and sugar-mediated signalling pathways, further indicated that both treatments resulted in cells experiencing a nutrient deficiency. Although we did not analyse cellular nutrient deficiency in the plants during our experiments, the profiles observed here are in agreement with existing information in annotation databases such as TAIR (release 10) and Gene Ontology, which are derived from the published literature.

Localised host programmed cell death is a crucial mechanism through which plants respond to pathogen and insect attack. This phenomenon regulates multiple physiological processes, including terminal differentiation, senescence, and disease resistance [37]. Several of the genes involved in the localised host programmed cell death categories were up-regulated during both treatments. These genes are also known to be induced by senescence and salicylic acid treatment, including the *PR* genes (PATHOGENESIS-RELATED GENE) *PR1, PR2, PR4* and *PR5*.

**Figure 2:**
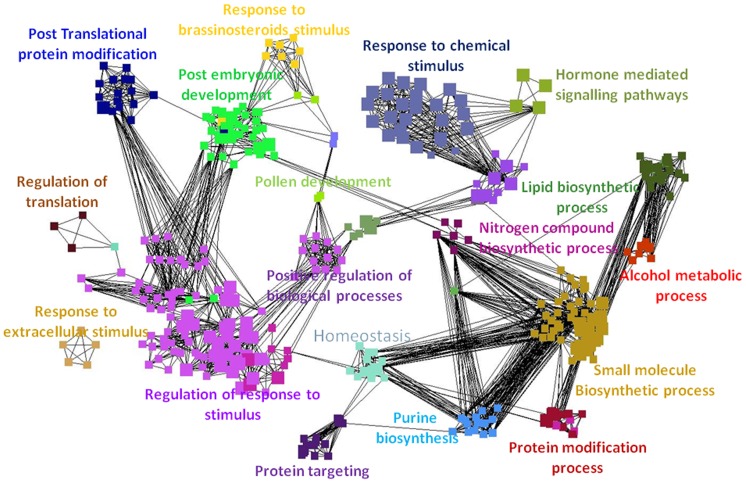
Over-represented GO-categories in the aphid-specific gene list. Network representations of enriched GO categories among genes that were differentially regulated only during the aphid experiment. Figure generated by ClueGO showing functionally grouped networks of enriched GO categories among genes whose expression was induced only in the aphid experiment. GO terms are represented as nodes based on their kappa score (≥0.3); only networks with at least three nodes are represented. The node size represents the significance of the term’s enrichment. The edges are related to the relationships between the selected terms, which are defined based on the genes that are shared in a similar way. The label for the most significant term is used as the leading group term. Visualisation was conducted using Cytoscape 2.7.0.

**Figure 3:**
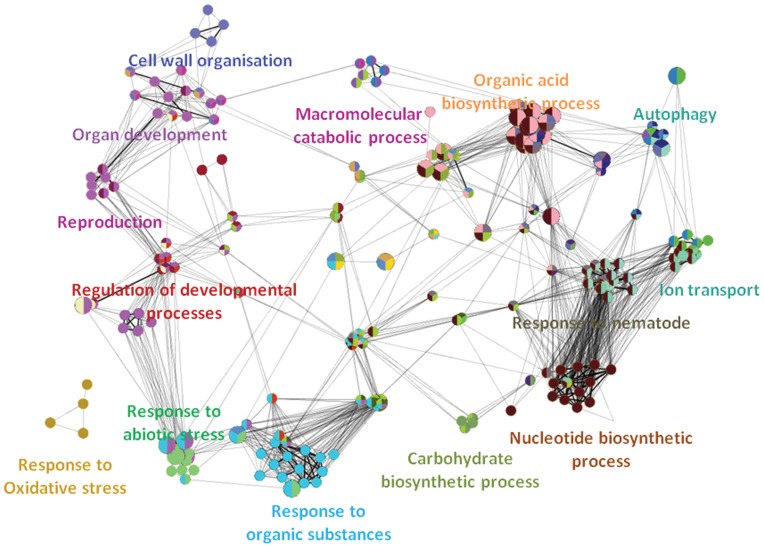
Over-represented GO-categories in the *P. syringae*-specific gene list. Network representations of enriched GO categories among genes that were differentially regulated only during the *P. syringae* experiment. Figure generated by ClueGO showing functionally grouped networks of enriched GO categories among genes whose expression was induced only in the *Pseudomonas* experiment. GO terms are represented as nodes based on their kappa score (≥0.3); only networks with at least three nodes are represented. The node size represents the significance of the term’s enrichment. The edges are related to the relationships between the selected terms, which are defined based on the genes that are shared in a similar way. The label for the most significant term is used as the leading group term. Visualisation was conducted using Cytoscape 2.7.0.

Visualisation of the networks of GO terms based on the aphid-specific responses **(** **Figure 2** **)** and *P. syringae*-specific responses **(** **Figure 3** **)** demonstrated the massive transcriptional responses evoked in *A. thaliana*. Most of the significant processes were related to responses to stimuli, biosynthesis of secondary metabolites, and transcriptional and posttranscriptional regulation. Superposition of the two GO term networks generated from the aphid-specific gene list and *P. syringae*-specific gene-list showed significant differences in the overrepresented GO terms. The superimposed network diagram has not been included in this manuscript, but all three networks (.cys file) have been provided as additional files **(files S1, S2,** and **S3)**. The interested reader can locally open these files in Cytoscape and conduct interactive exploration. (For local visualisation, download cytoscape software from http://www.cytoscape.org/, and load the.cys files on the software. Please note that the view of the annotated network presented in this manuscript has been manually simplified for representation purposes.).

### Mapping the Insect- and Bacterial-specific Responses on Pathways and Processes

To structure the genes present on the *A. thaliana* whole-genome microarray, they were assigned to functional categories using the pathway analysis program MapMan (http://gabi.rzpd.de/projects/MapMan, version 3.5.0). MapMan is a user-driven tool that displays large datasets such as gene expression data from Arabidopsis microarrays in diagrams of metabolic pathways or other processes. After the normalisation of expression values, differential fold-change values were calculated with statistical tests, as described in the Materials and Methods section. The ratios in the 4 biological replicates were averaged and converted to a log 2 scale, then imported into MapMan as ‘.xls’ files **(files S4, S5)**. MapMan converts the values to a false colour scale and displays them in diagrams. Transcripts that increase, decrease or change less than a given threshold are shown in blue, red and white, respectively. Some of the important categories (or functional BINs as per MapMan definition) identified via MapMan analysis are explained below.

### Metabolism Overview Map

**Figure 4:**
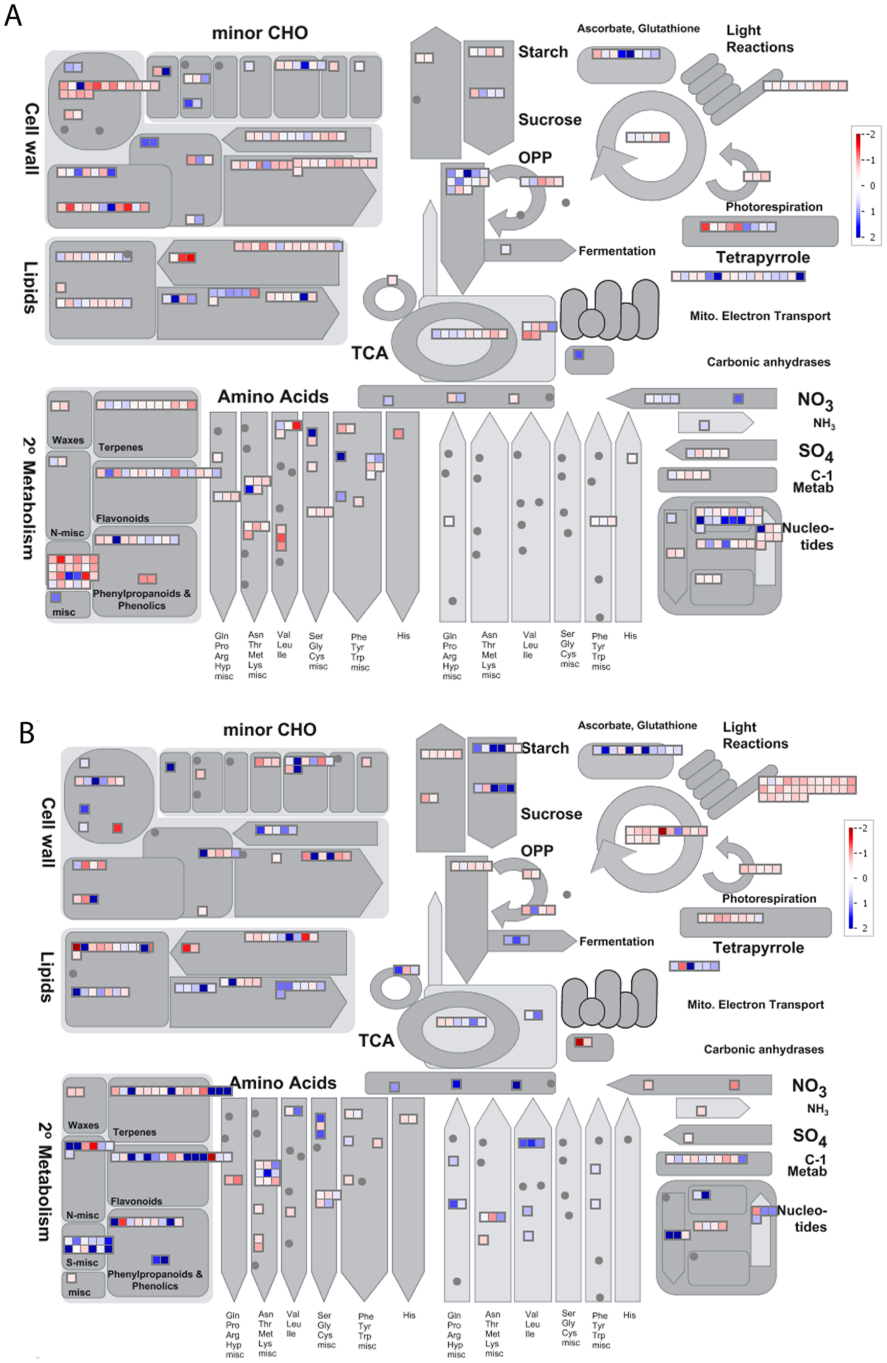
Metabolic overview map. Metabolic pathways associated with the transcriptional changes affecting *A. thaliana* during aphid *and P. syringae* attack. Overview of the expression changes related to metabolic pathways observed in *A. thaliana* plants during the (**A**) aphid and (**B**) *P. syringae* treatments using MapMan software. The represented spots are only for genes showing a significant (P = 0.01) change in expression between the treatment and the untreated control that were attributed to the respective bins by MapMan. Genes whose expression levels were increased are indicated with an increasingly blue colour, while decreasing expression is indicated in red. The graduation can be seen on the scale presented in the top right corner of each subfigure. A change in expression of log2 = 2.0 scale was selected as giving full saturation.

An overview of the transcriptional responses affecting genes coupled to metabolic processes showed that many genes connected to photosynthesis and energy metabolism were down-regulated after *P. syringae* and aphid attack **(** **Figure 4** **)**. *P. syringae* infection resulted in leaf senescence and leaf yellowing, which had a major effect on chloroplast function and processes connected to the chloroplast, such as fatty acid biosynthesis, carotenoid production, chlorophyll biosynthesis, carbon fixation and others. Genes related to these processes showed clear down-regulation following *P. syringae* treatment. Secondary metabolism was strongly affected during both treatments, particularly regarding the phenylpropanoid and glucosinolate pathways. The results of *P. syringae* treatment also showed that genes connected to the terpenoid and alkaloid pathways were up-regulated, including *DXPS1, TPS10, GES/TPS04, SS2, SQE6* and *LAS1*. In general, the stress associated with the activation and continuation of defence responses is metabolically expensive, and the plant must reallocate a significant amount of the resources that would normally be used in plant growth and reproduction to the production of defence-related compounds [38], [39]. However, in a recent work, Foyer et al. [40] explained that the decreases in growth and photosynthesis in response to stress are more likely the result of programmed down-regulation. Our experimental results showed that exposure to two biotic stresses resulted in the down-regulation of genes linked to auxin, gibberelin and cytokinin responses as well as genes coupled to cell wall modifications and cell division. The infected plants might also compensate for the depletion of sugars and amino acids, resulting in increased carbon assimilation and mobilisation of carbon, mannitol and nitrogen reserves. The plants may have degraded proteins/amino acids to generate energy (glycolysis) and re-assimilate nitrogen, through the glutamate dehydrogenase *GDH2* or lysine-ketoglutarate reductase (At4g33150). There were also genes connected to starch degradation/sugar responses induced, indicating that the plants might be degrading starches, e.g., *BAM5* and *GPT2*, used in glycolysis. Starch biosynthesis genes were generally down-regulated. The degradation of starch and maltose may also generate an osmotic force that balances water losses. During an aphid infestation, plants suffer from osmotic stress as the insect sucks large quantities of liquids from them. To counteract this situation, the transcription of genes involved in the regulation of water balance was observed to be induced, such as the *WRKY40*, *CYP707A3* (ABA-biosynthesis), *ZAT10* and *ZAT7.*

### Comparative Overview of the Response to Biotic Stress during the Aphid and *P. syringae* Treatments

**Figure 5:**
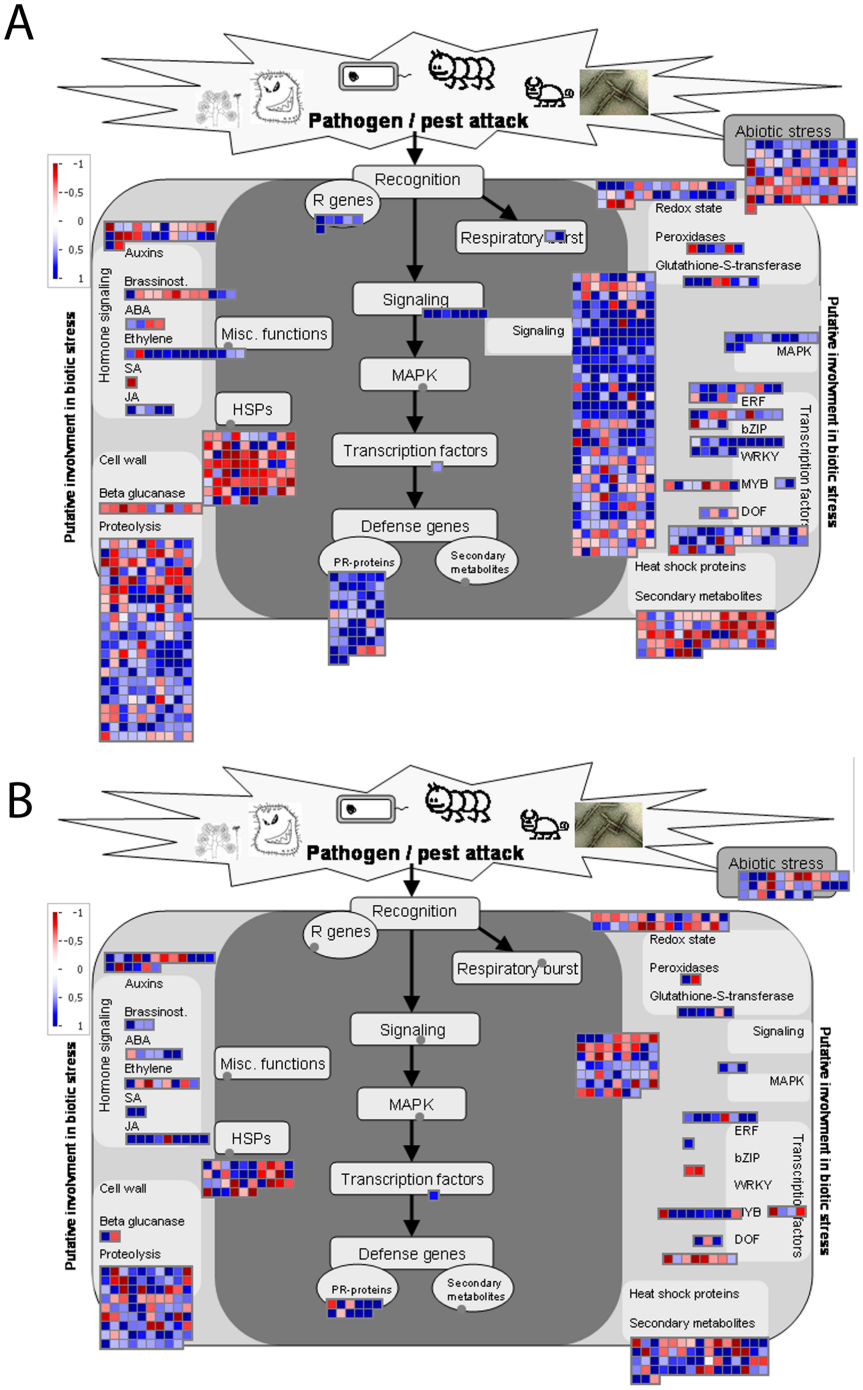
Biotic stress response overview map. This figure shows the changes in the expression of biotic stress-responsive genes in *A. thaliana* plants during the response to the aphid and *P. syringae* treatments. Genes that have been experimentally indicated to be involved in biotic stress are collected in the main panel (coloured with dark grey), while genes and pathways that are putatively involved in biotic stress pathways are shown on the left and right sides (coloured in light grey). (**A**) Aphid infestation. (**B**) *P. syringae* infection. In both cases, the signal after infection is expressed as a ratio relative to the signal in uninfected controls, which was converted to a log2 scale and displayed. The scale is shown in the figures. Only the genes showing a significant (P = 0.01) change in expression between the treatment and the untreated control that were attributed to the respective bins by MapMan are shown. Genes whose expression was increased are indicated with increasingly intense blue and red colours. The gradation can be seen in the scale presented in the top right corner of each subfigure.

A plant’s reaction to biotic stress involves several steps: after the initial signal input from the pathogen, which is recognised by the corresponding receptors (putative R genes), transcription of the cascade associated with the plant defence mechanism is triggered, including changes related to oxidative stress. Inside the cell, signals are transmitted and lead to the production of defence molecules (PR proteins, heat shock proteins and secondary metabolites). A large number of signalling genes were activated during both the aphid and *P. syringae* treatments **(** **Figure 5** **)**. Most of these genes encode receptor kinases, leucine-rich receptor kinases, MAP kinases, calcium-binding proteins and proteins regulating oxidative stress, such as peroxidases (details in **file** **S6**). The number of signalling proteins that were differentially expressed during the aphid experiment was more than four times higher compared to the *P. syringae* treatment. There were 278 aphid-specific signalling genes, but only 62 *P. syringae*-specific signalling genes. Thirty-one heat shock proteins were differentially expressed only during the aphid treatment (**file** **S7**), the majority of which were of the *DnaJ/Hsp40* type chaperones and were induced. Large numbers of proteolytic enzymes were differentially expressed during both the aphid (220) and *P. syringae* (89) treatments. The majority of these enzymes were ubiquitin proteases, F-box proteins, cysteine proteases, serine proteases, *C3HC4-type RING fingers*, and metalloproteases (**file** **S8**). Several of the down-regulated proteolytic enzymes were chloroplast localised or predicted to be located in the plastid/chloroplast, while most of the *C3HC4-type RING finger* proteins were induced. Secondary metabolites play a crucial role during plant defences. Sixty-three genes related to secondary metabolic processes were differentially regulated during the aphid and *P. syringae* treatments. Some of these secondary processes include the biosynthesis of isoprenoids, phenylpropanoids, glucosinolates and flavonoids. A detailed analysis of the differentially regulated secondary metabolic processes can be found in **file S9** and in a later section of this article. There were 76 differentially regulated genes connected to cell wall-related processes identified during the aphid treatment, but only 36 in the *P. syringae* experiment. These genes included components involved in cell wall precursor synthesis, cellulose synthases, cell wall structural proteins such as *AGPs* (arabinogalactan protein), *LRR* (leucine-rich repeat) extensin-like proteins, and *HRGPs* (hydroxyproline-rich glycoproteins) (details in **file S10)**. In general, aphid attack appeared to affect cell wall-related processes to a greater extent than *P. syringae* infection. In particular, a large number of *APGs* and xyloglucan:xyloglucosyl transferases were observed to be down-regulated. Xyloglucan endotransglucosylases are known to play an important role during cell elongation and cell wall modifications during shade avoidance [41], [42]. The effects observed on genes encoding pathogenesis-related proteins (*PR* proteins) showed a clear bias between the treatments: while only 11 *P. syringae-*specific *PR* proteins were affected, 56 genes encoding aphid-specific *PR* proteins showed differential expression. The *PR* proteins include a wide variety of protein types, such as ß-1,3-glucαnases, chitinases, thaumatin-like protein, proteinase inhibitors, plant defensins and others. The PR1 protein, which is often used as a marker for salicylic acid responses, was more than ten-fold higher induced by the aphid attack than by *P. syringae* infection. Another class of proteins that was induced and significantly overrepresented after aphid attack corresponded to a large number of disease resistance proteins belonging to the *TIR-NBS-LRR* (Toll/Interleukin1 receptor–nucleotide binding site–leucine-rich repeat) proteins.

Among the biotic stress-related transcription factors, some *WRKY* and *bZIP* proteins were expressed differentially only during the aphid experiment, while some *MYB* proteins were expressed differentially only during *P. syringae* infection. Other differentially regulated classes of transcription factors included *ERF/AP2, NAC, bHLH* and *DOF*. Details regarding the differentially regulated transcription factors are provided in a separate section of this article. The plant defence responses associated with *P. syringae* and aphid attack induced and repressed various hormonal signalling pathways. The most affected of these pathways during our experiments were the JA, SA, ABA, ethylene and auxin pathways. Among the hormonal signalling pathways, some components of the ethylene, JA, SA, ABA, auxin and brassinosteroid pathways appeared to specifically be regulated during the aphid and *P. syringae* treatments. There were relatively few ethylene responses observed in general, but such effects were clearly stronger after the aphid than the *Pseudomonas* treatment. Examples of ethylene responses included *ACS6, ERF11*, which may modulate ABA-regulated ethylene biosynthesis, *ORA59*, which integrates JA and ethylene signals during plant defence, *EFE* (ethylene forming enzyme) and *ATARD3* (methionine recycling during ethylene synthesis). Some proteins involved in the biosynthesis of ethylene were also affected. JA was more strongly induced by *P. syringae*, but the SA response was stronger following aphid attack. The details of the differentially regulated genes involved in hormone-mediated signalling pathways are provided in a separate section of this article.

### Regulatory Overview Map

**Figure 6:**
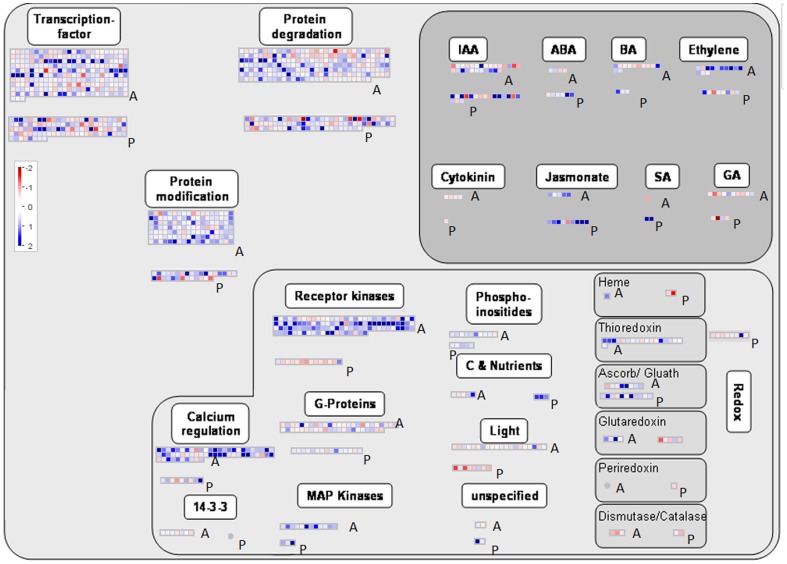
Regulatory overview map. MapMan regulatory overview map showing differences in transcript levels between aphid-specific and *P. syringae*-specific genes. Aphid-specific and *P. syringae*-specific bins are marked as ‘A’, and *P. syringae*-specific bins are marked as ‘P’. In the colour scale, blue represents higher gene expression, and red represents lower gene expression. IAA, Indole-3-acetic acid; ABA, abscisic acid; BA, brassinosteroid; SA, salicylic acid; MAP, mitogen-activated protein.

The categories that included most of the induced regulatory genes were TFs, receptor kinases, protein degradation and protein modification. In addition, several genes involved in overrepresented induced biological processes, such as the auxin signalling pathway and autophagy, were included in the regulatory categories **(** **Figure 6** **)**. A comparative list of the differentially expressed genes (both aphid-specific and *P. syringae*-specific genes) involved in hormonal pathways and their corresponding log2-transformed expression values are provided in **file S11**. The ethylene pathway was up-regulated after the aphid treatment, and genes such as *ACS6, ATARD3, ERS1* and a number of ethylene responsive element binding factors (*ERFs*) were induced. Genes belonging to the *ERF/AP2* family are induced by many biotic and abiotic factors, among which ethylene *ERFs* not only control a subset of ET-mediated responses but might also integrate ET with other signalling pathways. Increased ethylene production is a common defence response after herbivore attack and has been reported in several plant species [43]. Both ABA and JA responses were up-regulated by the *P. syringae* treatment, but few known SA-responsive genes were induced. Two categories, receptor kinases and calcium regulation (in **Figure 6**), appeared to be quite highly represented according to the MapMan annotation during the aphid experiment. Nevertheless, two other categories, light signalling and redox control, included fewer transcripts and gene families, respectively. These were some key differences between the aphid and *P. syringae* treatment. Genes encoding receptor kinases and proteins coupled to calcium signalling were overrepresented following the aphid treatment. These genes include a large number of cysteine-rich receptor-like protein kinases, such as, *CRK7, CRK37, CRK36, CRK23, CRK14, CRK11, CRK15, CRK6, CRK28* and calmodulin-like proteins (*CML40, CML47, CML11, TCH3, TCH2/CML24, CML44, CML45, CML30, CML37, CML38* and others) as well as several calmodulin-binding IQ-domain proteins. Together with the MAP kinases (*MPK11, MKK9, ATMPK3, MEKK3, MEK1, MEKK1, MKK2, MKK4, MPK4* and others), they constitute a large network that activates various plant defence responses, resulting in the activation of key transcription factors.

### Differences Observed in the Jasmonic Acid (JA) Biosynthesis Pathway during the Aphid and P. syringae Treatments

The jasmonic acid signalling pathway is a highly conserved, powerful regulator of plant defence signalling that is activated during infection by various pathogenic microorganisms as well as upon insect attack [44]. Kuśnierczyk et al. reported that more than 200 genes are dependent on the plant’s jasmonate status, irrespective of external stimuli, and that the aphid-induced response of more than 800 transcripts is regulated by jasmonate signalling [45]. The release of linolenic acid from membrane lipids initiates a series of enzymatic reactions known as the octadecanoid pathway, leading to accumulation of JA and related compounds. Additionally, 12-oxophytodieonic acid (*OPDA*) is a biosynthetic precursor of JA signalling molecules, which activate the expression of related-related genes. The selection of transcripts induced by JA and *OPDA* varies to some extent. This difference can be attributed to the electrophilic activities of the cyclopentanone ring of JA [46]. A number of enzymes coupled to oxylipin/JA biosynthesis, such as *AOC3, OPR3, OPCL1, LOX2* and *LOX3*, were up-regulated by both treatments, while *AOS, AOC1, AOC2, AOC4, ACX1, ACX5* and *LOX1* were mainly induced by *Pseudomonas*. Two *OPR*-related genes, At1g18020 and At1g17990, as well as the lipoxygenases *LOX4, LOX5* and *LOX6* were only induced by aphid attack. Almost none of the genes encoding proteins potentially linked to oxylipin biosynthesis were down-regulated, with the exception of *OPR1*, which was down-regulated by *P. syringae* infection.

### SA Regulates the Expression of Aphid-specific Defence Proteins, and Methyl Salicylate Activates *P. syringae*-specific Defence Proteins

Salicylic acid is another stimulator of plant defence responses and is an important trigger of systemic acquired resistance (SAR), resulting in increased defence against a variety of pathogens. Methyl salicylate (MeSA) has been identified as one of the mobile signals required for SAR. MeSA is translocated from the site of infection through the vascular system to distal (systemic) tissues, where it activates specific defence responses. The SAR response results in a complex chain of events and is regulated by various transcription factors. In higher plants, SA can be synthesised from phenylalanine via cinamic acid or from isochorismate. During pathogen attack, SA signalling leads to accumulation of various pathogenesis-related proteins (*PR* proteins), which can possess antimicrobial and anti-insect activities. Interestingly, MeSA released by the attacked plants can be detected by insects and changes their plant preferences [47]. In our analysis, expression of a methyltransferase gene (At3g21950) related to salicylate O-methyltransferases was down-regulated during aphid treatment. At3g21950 encodes a S-adenosyl-L-methionine:salicylic acid carboxyl methyltransferase related to BSMT1 that may convert SA to MeSA. In contrast, other methyltransferase genes BSMT1 (At3g11480), which converts SA to MeSA, and UGT74E2 (At1g05680) were up-regulated during *P. syringae* treatment **(file S11)**. UGT74E2 is hydrogen peroxide responsive and may be involved in water stress responses. There were relatively few known genes coupled to the biosynthesis of SA found in both datasets. However, BSMT1 might be a key enzyme.

Although relatively few genes connected to the biosynthesis of SA and MeSA were found in the obtained datasets, several genes induced by SA were identified. Additionally, isochorismate synthase 1 (*ICS1*) and one of its transcriptional regulators, *WRKY46*, were induced by aphid infestation. Another gene induced after aphid treatment that may be under the regulation of *WRKY46* is *PBS3*. *PBS3* most likely encodes an enzyme producing SA-glucoside, a putative storage form of SA, and *pbs3* mutant plants exhibit impaired activation of defence genes such as *PR1*. The PR1 gene, a common marker for SA-induced genes, was strongly up-regulated by the aphid treatment (log2 = 5.5) and slightly less induced by *P. syringae* treatment (log2 = 1.7). The *WRKY53* gene, which is known to be up-regulated by SA [48], was only induced in the aphid treatment. A number of genes, such as *ALD1* and *BAP1*, coupled to systemic defence responses were uniquely induced by aphids.

### Overview of Differences in Secondary Metabolism

Plants have evolved many secondary metabolites involved in plant defence, which are collectively known as antiherbivory compounds and can be classified into three sub-groups: nitrogen compounds (including alkaloids, cyanogenic glycosides and glucosinolates), terpenoids, and phenolics [49]. In addition to the three larger groups of substances mentioned above, fatty acid derivatives, amino acids and even peptides are used in defence. The terpene synthase genes *GES* (geranyllinalool synthase, At1g61120), *LAS1* (Lanosterol synthase, At3g45130), and *TPS10* (Terpene Synthase 10, At2g24210) were highly up-regulated during P. syringae treatment. The elicitor-activated gene *CAD-B2* (At4g37990), belonging to the phenylpropanoid metabolism category, was strongly up-regulated in a *P. syringae*-specific manner. In the alkaloid-like compound biosynthesis category, strictosidine synthase genes (At1g74010, At1g74020) were highly up-regulated in the *P. syringae* experiment. In the flavonoids category, two genes *SRG1*(Senescence-Related Gene 1; At1g17020) and 2-oxoacid-dependent oxidas (At3g50210), were also up-regulated in a *P. syringae-*specific manner. Significant differences were observed in genes coupled to glucosinolate biosynthesis and hydrolysis. Glucosinolates (*GS*) are secondary metabolites typical of the order *Brassicales* [50]. Most of the aphid-specifically expressed aliphatic *GS* genes were repressed, whereas most of the *Pseudomonas*-specifically expressed genes were positively regulated. The lists of these genes are provided in **Tables 2** and **3** **.** Following *P. syringae* treatment, two myrosinase-associated proteins (At1g52040, At1g54020) and a nitrile-specific protein *AtNSP5* (At5g48180) were highly up-regulated. It was reported by Kissen *et al.,* that the nitrile specifier proteins involved in glucosinolate hydrolysis in *Arabidopsis thaliana* and products generated after hydrolysis, such as isothiocyanates, play multiple roles in growth regulation and defence [51].

**Table 2:** Genes involved in glucosinolate metabolism affected by aphid infestation.

Gene ID	Log2	Description
At1g52040	4.185	*MBP1*
At1g54020	3.094	myrosinase-associated protein, putative
At5g48180	2.293	*NSP5*
At3g19710	1.231	*BCAT4* (branched-chain aminotransferase4)
At1g16400	0.72	*CYP79F2*
At1g62540	0.696	*FMO GS-OX2*
At5g25980	0.487	*TGG2, BGLU37*
At4g13430	0.448	*IIL1*
At2g44490	−0.675	*PEN2, BGLU26* (penetration 2)
At1g54010	−0.2	myrosinase-associated protein, putative
At1g62570	1.56	*FMO GS-OX4*

**Table 3:** Genes involved in glucosinolate metabolism affected by *P. syringae* infection, with log2 fold-change values.

Gene ID	Log2	Description
At4g03070	−0.869	*AOP1, AOP, AOP1.1*
At3g49680	−1.529	*ATBCAT-3*
At3g58990	−0.648	aconitase C-terminal domain-containing protein
At2g43100	−1.12	aconitase C-terminal domain-containing protein
At1g80560	−0.7	3-isopropylmalate dehydrogenase
At1g31180	−1.045	3-isopropylmalate dehydrogenase
At4g13770	−0.415	*CYP83A1, REF2*
At2g31790	−0.813	UDP-glucoronosyl/UDP-glucosyl transferase family protein
At1g18590	−0.679	*SOT17, ATSOT17, ATST5C*
At1g74090	−0.901	*SOT18, ATSOT18*
At1g12140	0.313	*FMO GS*
At1g65860	−0.97	*FMO GS-OX1*
At1g62560	−0.679	*FMO GS*
At4g03060	−1.248	*AOP2* (alkenyl hydroxalkyl producing 2)
At5g57220	1.527	*CYP81F2*
At4g31500	1.352	*CYP83B1, SUR2, RNT1, RED1, ATR4*
At5g07690	−1.511	*MYB29, ATMYB29, PMG2*
At5g61420	−0.707	*MYB28, HAG1*
At2g33070	0.524	*NSP2*
At4g12030	−0.882	sodium symporter family protein

### A Large Number of Transcription Factors are Differentially Regulated, Many of which are Unique to Insect or Bacterial Stress

**Figure 7:**
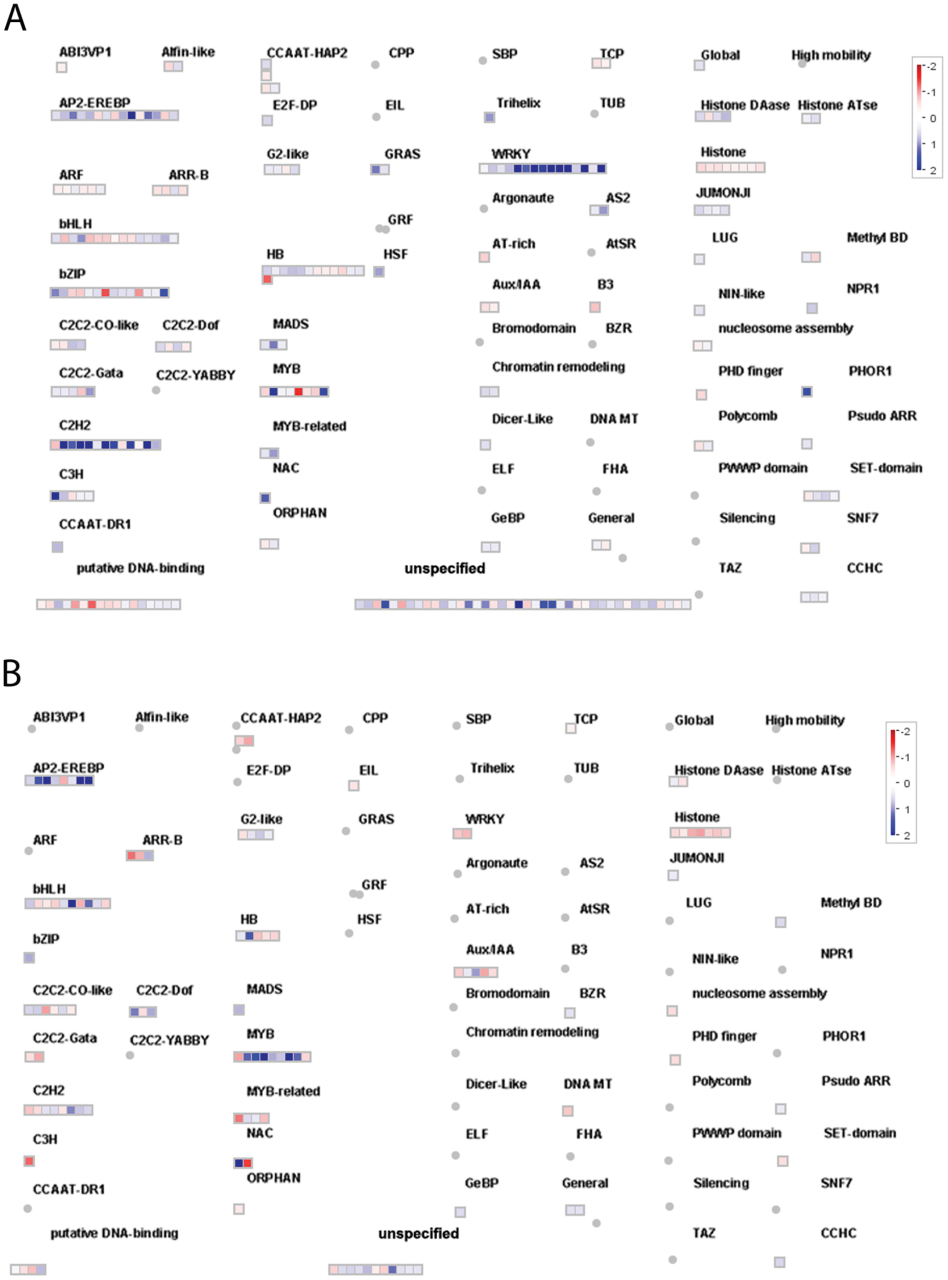
Transcription overview map. (A) Aphid specific; (B) *P. syringae* specific.

Transcription factors are the key regulators of gene expression changes and, thus, represent important part of a complex regulatory network allowing plants to adjust to changes in their environment [52]. Members of several Arabidopsis transcription factor families have been linked to plant stress responses, and a significant overlap in the expression profiles of many of these genes corresponding to a range of stress conditions has been reported. TFs are often induced by signalling phytohormones such as JA, SA or ET. The TFs that were differentially expressed during the aphid and *Pseudomonas* treatments are reported in **Table 1**, and their names are given in **file S12**. Additionally, pictorial representations of the aphid-specific and *P. syringae*-specific TFs produced using MapMan software are shown in **Figure 7**. There were 16 WRKY TFs that were up-regulated in an aphid-specific manner (*WRKY20, WRKY22, WRKY39, WRKY21, WRKY40, WRKY26, WRKY50, WRKY25, WRKY38, WRKY51, WRKY53, WRKY47, WRKY46, WRKY69, WRKY33, WRKY16*). *WRKY* TFs can act as both positive and negative regulators of plant defence pathways. The mechanisms activating *WRKY* TFs can involve the MAP kinase cascade and calcium signalling. It has been demonstrated that a subgroup of *WRKY* TFs can act as calcium concentration sensors, being activated by the increase in the Ca^2+^ concentration that occurs under inducer attack [53]. Mechanical penetration of cells by aphid stylets changes the plasma membrane potential and increases in intracellular Ca2^+^ concentrations. Fluctuations in the cytosolic Ca^2+^ concentration resulting from the opening of membrane-bound calcium channels are further decoded by several Ca^2+^-binding proteins, including the *WRKY* TFs. The up-regulated aphid-specific TFs also include C2H2 zinc finger proteins.

The MYB family, which is another large family of TFs characterised by a conserved MYB DNA-binding domain, bind to a variety of different DNA sequences. Among the *P. syringae*-specific TFs, there are 9 *MYBs* (*MYB95, MYB112, MYB90, MYB102, MYB32, MYB114, MYB59, MYB60, MYB20)*, of which the first 7 are significantly up-regulated. *MYB90* is also known as *PAP2* (PRODUCTION OF ANTHOCYANIN PIGMENT 2), suggesting that some of these MYBs are likely to be involved in anthocyanin biosynthesis. A few members of this family specifically activate genes related to tryptophan aliphatic glucosinolate and indoyl glucosinolate synthesis [54].

### Integrated Information from Available Public Domains Reveals a Pattern of Potential MicroRNA-mediated Post-transcriptional Regulation during Insect and Bacterial Attack

We constructed a genetic network of the differentially regulated gene lists using the *Gene network* tool in *VirtualPlant*. First, individual genes belonging to the common category were grouped into a “super node” based on shared functional properties, such as GO terms, KEGG pathways, Gene families and even similar annotations. The functional annotations were categorised in a hierarchical manner, where the functional terms and pathways were themselves grouped into higher, more generic categories (details are given in the Materials and Methods). During this analysis, we used post-transcriptional regulation, protein-protein interactions, and transcriptional regulation information from both experimental and predicted databases. The ‘Regulated Edges’ are predicted interactions based on the presence of known transcription factor cis-acting binding sites located in the 3 kbp upstream region of annotated transcripts. Interestingly, some of the key stress-regulated transcription factors are reported in publications, or have been computationally predicted to be regulated by different microRNAs. Thus, we were able to hypothesise that the activation of microRNA genes under biotic stresses would lead to the repression of many downstream protein-coding genes and affect physiological responses. This analysis indicates a new direction for conducting large-scale experiments and subsequent bioinformatics analyses to explore the regulatory links between biotic stress and microRNAs in *A. thaliana*.

### Connection of microRNAs to Genes from the Common Category

**Figure 8:**
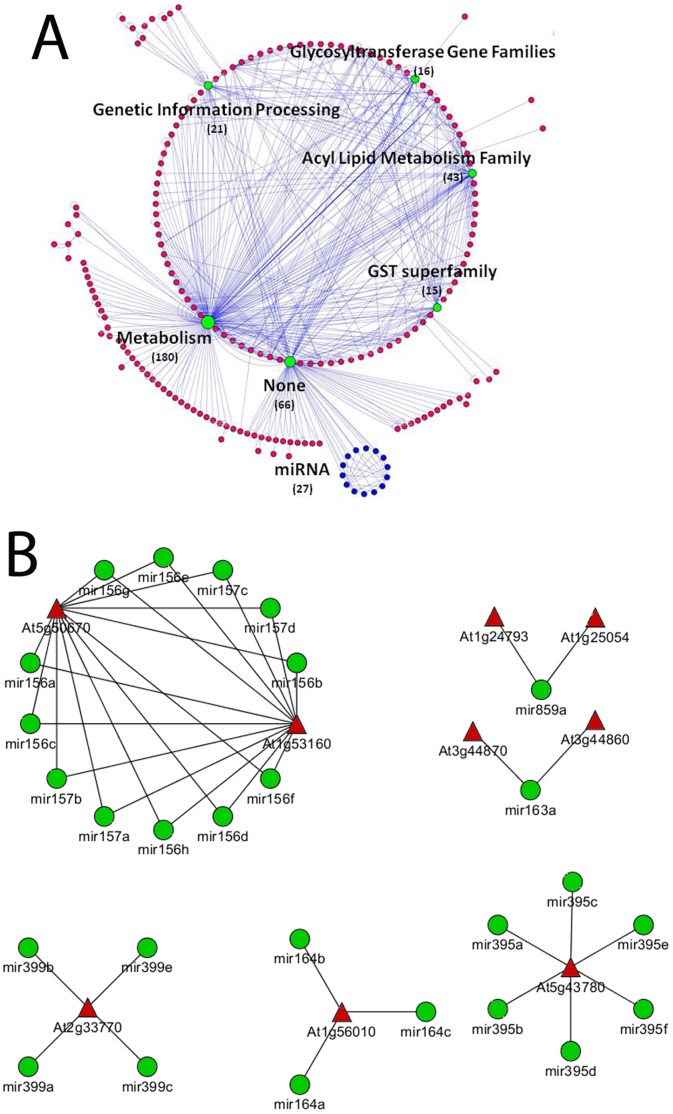
Retrieved micro-RNA connections of the common genes. **A)** Super node analysis using the *Gene networks* tool in *VirtualPlant*, visualised with Cytoscape 2.7.0. Individual genes in the common category were grouped into a supernode (red-coloured nodes) based on shared functional properties, such as GO terms, KEGG pathways, gene families and even similar annotations. Each supernode size corresponds to the number of genes present in that category. The edges represent connections among different functionally grouped supernodes. The top 6 most highly populated supernodes are filled with green colour. A supernode consisting of 66 genes known to show connections with 27 microRNAs (cluster of blue-coloured nodes). microRNA binding sites have been reported in existing literature for 9 of these genes, and 6 of them are known transcription factors. **B)** Details of the 9 genes mentioned above, which are known to be regulated by 27 microRNAs. MicroRNAs are shown as green-coloured circles, whereas target genes are depicted as red-coloured triangles. Edges represent the interactions between microRNAs and their target genes. Please also refer to **Table 4** and **Table 5** for detailed information and related evidence in the literature.

Supernode analysis of the differentially expressed common genes using the *VirtualPlant* tool revealed a supernode, or cluster, of 66 genes known to show connections with 27 microRNAs **(** **Figure 8A** **,** marked as a blue-coloured cluster**).** Further analysis on this cluster of 66 genes identified 9 genes (**Table 4**) with experimentally validated microRNA-binding sites **(** **Figure 8B** **)**. Six of these genes encode known Arabidopsis transcription factors. Manually retrieved related literature references for each of this microRNA are provided in **Table 5**. We then studied all of the microRNA genes curated in the microRNA Registry database (microrna.sanger.ac.uk/sequences/). Out of the 66 genes in this cluster **(** **Figure 8A** **,** supernode annotated as ‘none’**)**, At1g20510 (*OPCL1*) and At4g05160 (putative 4-coumarate-CoA ligase/4-coumaroyl-CoA synthase) are known to be involved in jasmonic acid biosynthetic processes. Two genes, At1g50670 and At5g53160 (*SPL4*), showed the maximum number of connections to microRNAs. *RD26* (RESPONSIVE TO DESSICATION 26, At4g27410), *BZIP25* (BASIC LEUCINE ZIPPER 25, At3g54620), *JIN1* (JASMONATE INSENSITIVE 1, At1G32640) and *BGLU11* (BETA GLUCOSIDASE 11, At1g02850) contain putative microRNA binding sites, though they have not yet been verified experimentally. Out of these 13 genes, 6 are known to be TFs. Details are given in **Tables 4** **and** **5** **.**

**Table 4:** The 9 genes in the common set known to be regulated by biotic stresses and their association with stress-inducible microRNAs (Refer to **Figure 8** **B**).

Gene ID	microRNA
At1g53160	*mir156f, mir156d, mir156h, mir157a, mir157b, mir156c, mir156a, mir156g, mir156e, mir157c, mir156b*
At5g50670	*mir156f, mir156d, mir156h, mir157a, mir157b, mir156c, mir156a, mir156g, mir156e, mir157c, mir156b*
At3g44860	*mir163a*
At3g44870	*mir163a*
At1g56010	*mir164b, mir164c,mir164a*
At5g43780	*mir395a, mir395b, mir395c, mir395d, mir395e, mir395f*
At2g33770	*mir399a, mir399b, mir399c, mir399e*
At1g24793	*mir859a*
At1g25054	*mir859a*

References from the literature related to each of the reported microRNA families are provided in **Table 5** **.**

**Table 5:** Functional targets of the microRNA families in the common set of genes (retrieved from literature searches).

microRNA	Target family
mir156 [86]	*SPL* family members, including *SPL3, SPL4*, and *SPL5*. By regulating the expression of *SPL3* (and probably also *SPL4* and *SPL5)*, this microRNA regulates vegetative phase change.
mir157 [87] [88]	*SPL* family members, including *SPL3, SPL4*, and *SPL5*.
mir163 [89]	*SAMT* family members. *mir163*, is highly expressed in *A. thaliana* diploids but down regulated in *A. thaliana* autotetraploids and repressed in *A. arenosa* and *A. suecica*.
mir164 [90]	*NAC* domains including *NAC1* and *ORE1*. Over expression leads to decreased *NAC1* mRNA and reduced lateral roots. Loss of function mutants have increased *NAC1* and increased number of lateral roots. Also targets *CUC2* and modulates the extent of leaf margin serration. Also targets *ORE1* to negatively regulate the timing of leaf senescence.
mir395 [91]	*APS* and *AST* family members.
mir399 [87], [88]	*PHO2*, an *E2-UBC* that negatively affects shoot phosphate content.
mir859 [92]	*F-box* family members.

### Connection of microRNAs to Genes Showing Aphid-specific Responses

**Figure 9:**
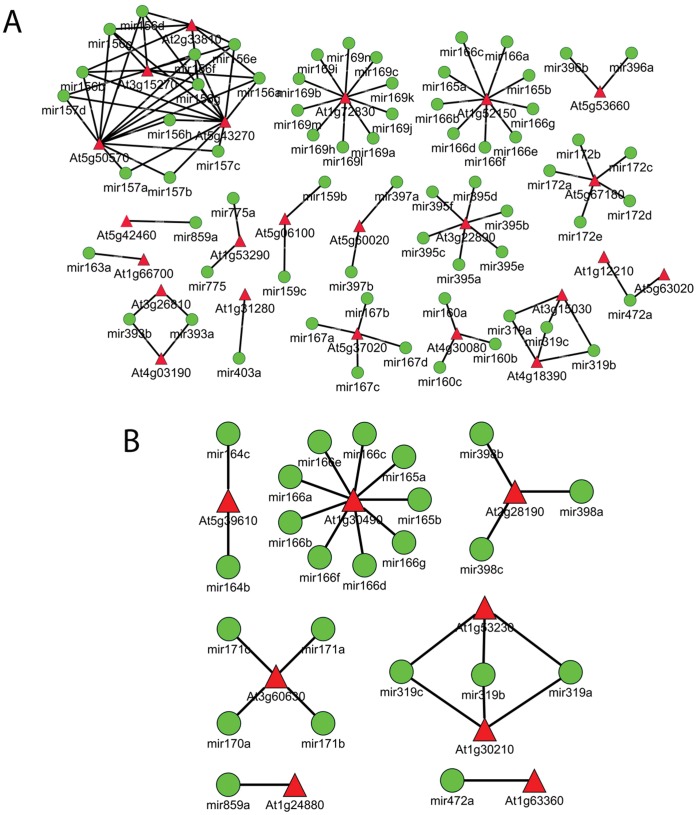
Retrieved micro-RNA connections of aphid-specific and *P. syringae*-specific genes. A red triangle represents a target gene, and a green circle represents a microRNA. A) Among the transcripts showing aphid-specific responses, 42 genes are known to contain microRNA binding sites. Please also refer to Table 6 and Table 7 for detailed information and related evidence from the literature. B) Among the transcripts showing *P. syringae*-specific responses, 9 genes are known to contain validated microRNA binding sites. We were able to find related references in the literature for the reported 23 microRNAs. Please also refer to Table 8 and Table 9 for detailed information and related evidence from the literature.

Among the 3,382 transcripts showing an aphid-specific response, the GO enrichment category ‘response to stimuli (biotic and abiotic stress)’ included 242 stress-responsive genes. Among these genes, 42 are known to exhibit transcription factor activity **(** **Figure 9A** **).** Additionally, out of these 242 stress-regulated genes, 21 genes have been reported to be associated with microRNAs based on literature and database searches, as described in the Materials and Methods section **(** **Table 6** **).** The reported target gene families for these microRNAs were retrieved through a manual literature search and are listed in **Table 7**. Many of the genes that were differentially regulated by aphid attack belong to these reported gene families.

**Table 6:** The 21 genes in the aphid-specific gene set known to be regulated by biotic stresses and their association with stress-inducible microRNAs.

Gene ID	microRNA
At3g15270	*mir156b, mir156f, mir156g, mir156a, mir156e, mir156d, mir156c, mir157d*
At2g33810	*mir156d, mir156c, mir156b, mir156f, mir156g, mir156a*
At5g43270	*mir157c, mir156h, mir157b, mir157a, mir157d, mir156c, mir156d, mir156b, mir156f, mir156e, mir156a, mir156g*
At5g50570	*mir157c, mir156h, mir157a, mir157d, mir156c, mir156d, mir156b, mir156f, mir156e, mir156a, mir156g*
At5g06100	*mir159b, mir159c*
At4g30080	*mir160a, mir160b, mir160c*
At1g66700	*mir163a*
At1g52150	*mir166a, mir166b, mir166c, mir165a, mir166d, mir166f, mir166e, mir166g, mir165b*
At5g37020	*mir167a, mir167b, mir167c, mir167d*
At1g72830	*mir169a, mir166b, mir16c, mir169m, mir169h, mir169l, mir169j, mir169k, mir169n, mir169i*
At5g67180	*mir172a, mir172b, mir172c, mir172d, mir172e*
At3g15030	*mir319a, mir319b, mir319c*
At4g18390	*mir319a, mir319b, mir319c*
At3g22890	*mir395a, mir395b, mir395c, mir395d, mir395e, mir395f*
At5g53660	*mir396a, mir396b*
At5g60020	*mir397a, mir397b*
At1g31280	*mir403a*
At1g12210	*mir472a*
At5g63020	*mir472a*
At1g53290	*mir775, 775a*
At5g42460	*mir859a*

Data retrieved from searches of the published literature and databases. (Refer to **Figure 9A**). References from the literature related to each of the reported microRNA families are provided in **Table 7** **.**

**Table 7:** Functional targets of the microRNA families in the aphid-specific set of genes (retrieved from the existing literature).

Micro-RNA	Target Gene family
mir156 [93]	*SPL2, SPL3, SPL4, SPL10*
mir157 [93]	*SPL* family members, including *SPL3,4,* and *5*
mir159 [94], [95]	*MYB* 107, *MYB* 116, *MYB33, MYB65, TCP2, TCP3, TCP4, TCP10, TCP24*
mir160 [93]	*ARF* family members (*ARF10, ARF16, ARF17*)
mir163 [96]	*SAMT* family members. *miR163*, is highly expressed in A. thaliana diploids but down-regulated in *A. thaliana* autotetraploids and repressed in *A. arenosa* and *A. suecica*.
mir165 [97]	*HD-ZIPIII* family members including *PHV, PHB, REV, ATHB-8, and ATHB-15*
mir166 [97]	*HD-ZIPIII* family members including *PHV, PHB, REV, ATHB-8, and ATHB-15*
mir167 [93]	*ARF* family members *ARF6* and *ARF8*.
mir169 [93]	*HAP2* family members
mir172 [94]	several genes containing *AP2* domains
mir319 [97], [98]	*TCP* family members.
mir395 [97]	*APS* and *AST* family members.
mir397 [97], [99]	targets several Laccase family members
mir403 [99]	*AGO2* and *AGO3*
mir472 [100]	Several *CC-NBS-LRR* family members.
mir859 [92]	Several *F-box* family members.

Most, but not all were affected by the aphid treatment.

### Connection of microRNAs to Genes Showing *P. syringae*-specific Responses

Among the 1602 transcripts showing *P. syringae*-specific responses, the GO enrichment category ‘response to stimuli (biotic and abiotic stress)’ included 146 genes. Out of these 146 stress-responsive genes, 24 are known to exhibit transcription factor activity **(** **Figure 9B** **)**, and 6 have been reported to be associated with microRNAs based on literature and database searches **(** **Table 8** **).** The reported target gene families for these microRNAs were retrieved through a manual literature search and are listed in **Table 9**. Many of the genes that were differentially regulated by *Pseudomonas* attack belong to these reported gene families.

**Table 8:** The 6 genes in the *Pseudomonas*-specific gene set known to be regulated by biotic stresses and their association with stress-inducible microRNAs.

Gene ID	microRNA
At1g30490	*mir165a, mir165b, mir166a, mir166b, mir166c, mir166d, mir166e, mir166f, mir166g*
At1g30210	*mir319a, mir319b. mir319c*
At1g53230	*mir319a, mir319b. Mir319c*
At2g28190	*mir398a, mir398b, mir398c*
At1g63360	*mir472a*
At1g24880	*mir859a*

Data retrieved from searches of the published literature and databases. (Refer to **Figure 9B**). References from the literature related to each of the reported microRNA families are provided in **Table 9**.

**Table 9:** Functional targets of the microRNA families in the *P. syringae*-specific set of genes (retrieved from the existing literature).

microRNA	Target Gene family
mir165 [51]	*HD-ZIPIII* family members including *PHV, PHB, REV, ATHB-8*, and *ATHB-15*
mir166 [97]	*HD-ZIPIII* family members including *PHV, PHB, REV, ATHB-8,* and *ATHB-15*
mir319 [51], [52]	*TCP* family members.
mir398 [97], [101]	*CSD* and CytC oxidase family members.
mir472 [53]	Several *CC-NBS-LRR* family members.
mir859 [46]	Several *F-box* family members.

Most, but not all were affected by the *Pseudomonas* treatment. Corresponding AtIDs are provided in **Table S6.**

### Cross-validation of Differentially Regulated Aphid and *Pseudomonas*-specific Transcription Factors via Co-expression Analysis of the Multiple Biotic Stress Dataset

**Figure 10:**
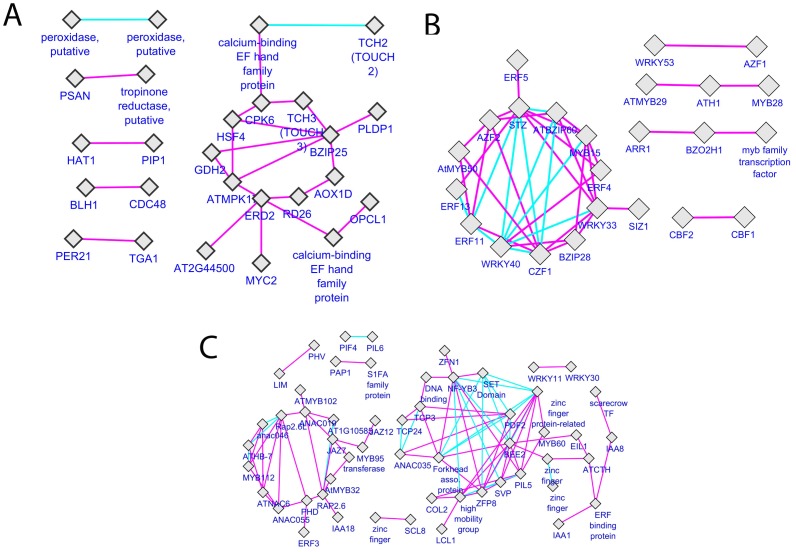
Co-expression network. Co-expression networks generated by CORNET using AtGenExpress biotic stress compendia based on a Pearson’s correlation coefficient threshold ≥0.7. The networks were visualised using Cytoscape2.7.0. Pink-coloured edges represent a strong correlation of ≥0.9, and cyan-coloured edges represent a correlation of ≥0.7 to 0.9. **A)** Co-expression network analysis among the 66-supernode cluster in the common group resulted in a network of 26 nodes 25 edges. **B)** Co-expression network analysis of the aphid-specific TFs resulted in a network of 24 tightly co-expressed TF modules. **C)** Co-expression network analysis among 104 Pseudomonas-specific TFs resulted in a tightly co-expressed modular network consisting of 55 nodes and 94 edges.

The differentially regulated gene sets included many signature transcription factors known for their involvement in stress responses. A co-expression analysis based on a compendium of 69 ATH1 biotic stress experiments, generated using the CORNET tool, showed that many of these TFs have been found to be strongly co-expressed during various biotic stress experiments. From the 66-gene supernode cluster in the common group, the co-expression analysis produced a network of 26 nodes with 25 edges **(** **Figure 10A** **)**. One module consisted of 9 genes *CPK6, TCH3, BZIP25, AOX1D, RD26, ERD2, MPK1, GDH2* and *HSF4* that were strongly co-expressed. The extended module contained 16 genes, 5 of which are involved in calcium-mediated signalling: *CPK6, TCH2, TCH3* and two EF-hand proteins. Functional annotation revealed that these genes are known to be involved in several different biotic and abiotic stress responsive processes. The calcium-dependent protein kinase (*CPK6*) is a positive regulator of methyl jasmonate signalling in guard cells and represents an important gene involved in methyl jasmonate signalling and signal crosstalk between methyl jasmonate and abscisic acid in guard cells [55]. *TCH3* is a calmodulin-like protein that is up-regulated in response to various environmental stimuli, including mechanical stimuli [56]. Responsive to desiccation 26 (*RD26)* encodes an *NAC* transcription factor that may be coupled to an ABA-dependent stress-signalling pathway [57], while the heat shock protein-70 cognate protein Early-responsive to dehydration (*ERD2*) which is induced by heat and dehydration is a key element in defence response signalling pathways [58]. The MAP-kinase gene *MPK1* participates in pathogen signalling, and its kinase activity increases in response to mechanical injury [59]. Glutamate dehydrogenase 2 (*GDH2*), the alpha-subunit of glutamate dehydrogenase, is a mitochondrial protein that has been reported to be responsive to diverse environmental stresses [60]. Arabidopsis heat shock factor (*HSF4*) regulates the expression of heat shock proteins [61]. The genes in the aphid-specific and pseudomonas-specific co-expression module have been discussed in previous sections.

### Conclusions

We generated and analysed data from two different biotic stress experiments conducted in *Arabidopsis thaliana* in which the plants were challenged with the aphid *Brevicoryne brassicae* and the bacterium *P. syringae syringae*. Our data showed that the transcriptional response of *Arabidopsis* to these very different attackers resulted in the differential regulation of a diverse range of biological processes. Transcriptional responses and networks unique to insect or bacterial stress conditions were identified, as were sets of genes showing similar a response under both stresses. By examining the responding genes and the functional network characteristics of each stress response, we found that a significant number of the transcripts encode transcription factors. Most of these transcription factors have shown to be involved in stress responses and regulatory processes. Some *WRKY* and *bZIP* genes were expressed differentially only during the aphid experiment, whereas some *MYB* genes were expressed differentially only during *P. syringae* infection. A Gene Ontology-based overrepresentation analysis revealed that half of the genes from the common list were involved in central metabolic and cellular processes, such as electron transport and energy pathways localised to the plastid. Secondary metabolism was strongly affected during both treatments, particularly the phenylpropanoid and glucosinolate pathways. Processes connected to the chloroplast, such as fatty acid biosynthesis, carotenoid production, chlorophyll biosynthesis, carbon fixation and others were down-regulated following *P. syringae* treatment. Starch biosynthesis genes were generally down-regulated, and an indication was found that the plants were degrading starch, which could help the plants to maintain the osmotic balance. Components of the ethylene, JA, SA, ABA, auxin and brassinosteroid pathways appeared to be specifically regulated during the aphid and *P. syringae* treatments. Ethylene responses were clearly induced during aphid feeding, while JA was more strongly induced by *Pseudomonas.* The number of signalling proteins that were differentially expressed during the aphid experiment was more than four times higher compared to the *P. syringae* treatment. By integrating secondary information from most available public sources, we further explored the regulatory links between biotic stress and microRNAs associated with aphid-and *P. syringae* -specific differentially regulated processes in *A. thaliana*, and the corresponding genes are briefly summarised in **Table 10** **.**

**Table 10:** Summary of aphid-specific and *P. syringae*-specific genes associated with differentially regulated processes during both of the treatments.

Categories	Aphid specific	*Pseudomonas* specific
Biotic stress signaling processes (up)	*FRK1, ATMPK11, ATRABA1e, PBP1, CRK11, EDA39, CRK6,CRT3, RLK5, LECRK1, ACA2, WAKL2,* *GLR2.7, ATMKK9, CML39, AP4.3A, CPK10, AtRABH1c, B120, MKK4, CPK29, ACA11, XLG2, CPK32,* *WAK2, RPK1, CPK7, ATSERK5, AGG1, NMAPKK, CPN1, RLK, CPK5, EP1, WAK1, ATHRGP1,* *WAKL22, MEKK1, CPK4, PHYD, CAM2, MKK2, AGG2, MPK4, ARK2, ELK4, FRS2,ATVPS34, RAN1,* *AtRABH1a, ARK3, RHA1, FRS5, CPK1, CAM3, CPK3, EFR, GRF1, MKK5, MSS3, MPK1, SOS3,* *ATGB1, AHP5, CRT1, CAM9, ATRABA1D, ATMPK15, MEE62, ATGDI1, PAT1, PIP5K9, ATPERK1,* *GLR1, APKKK5, WAKL6, GRF6, ATGDI2, BON2, GRF5, JAB1, GRF10, RAB6A,* *ARG, SIRANBP, ATG5, TIC, RAN3*	*AHP1, ATCP1, ATPH1, ATRABA1A, AtRABA1g, AtRABA5d, ATRABC2B, ATRABD2B, AtRABE1a, ATSARA1A, CCL, DRP1A, GLR1.2, GLR1.3, IQD14, MAPKKK18, MAPKKK3, MPK7 MSL4, PHYB, PLC1, RABF1, RD20, SAC9, SMG1*
Biotic stress signaling processes (down)	*AtRABA1f, AtRABA2d, AtRABA5b, ATRABE1C, ATRABE1D, AtRABG3d, CAM7, CPK8, ECT1, FRS12, GLR3.6, GRF2, GRF4, HSL1, iqd21, IQD31, LRR1, LSH1, MSL6 NIK1, NPGR1, NPGR2,NPY1, PAP2,* *PHYC, PHYE, RALFL22, RALFL23, RCI1, ROPGEF1, RPT1, SCABP8, SnRK1.2, SPA1, TOC33,* *VAN3, VAR3*	*ACA4, ATCAMBP25, ATRABC2A, BAM1, iqd2, IQD3, NIK3, PKS1, QRP1, RABG3B, RALFL32, SRL2, TMK1*
HSPs (up)	*ATJ1, ATJ2, ATJ3, BIP1, BIP2, BIP3, HSF A4A, HSP70, HSP70-1, HSP81-2, HSP81-3, HSP83,* *HSP91, J8, KAM2, MTHSC70-2, SHD*	*–*
HSPs(down)	*ARL1*	*–*
Proteolyitc enzymes(up)	*ATAPG9, AtATG18d, AtATG18f,ATL2, ATL6, ATL8, AtMC2, AtMC3, AtMC4, AtPNG1, AtPP2-B10, ATTLP9, BCS1, bt5, DA1, EBF2, FUS9, MCP1B, mos5, NHL8, PAC1, PAE2, PAF1, PBC2, PBG1,* *RHA1A, RHC1A, RHF2A, RKP, RMA1, scpl46, SKIP4, SUMO3, UBC15, UBC18, UBC23, UBC25,* *UBC33, UBC35, UBC9, UBP22, UBP3, UBP4, UBP5, UBP9, UBQ11, UEV1B, UPL3,* *UPL6, XERICO*	*AIR3, ATAPM1, ATG5, ATG8A, ATG8F, ATG8I, ATGGH1, ATGGH2, ATGGH3, AtTLP7, BPM2, HSP93-V, NSF, PAA1, PBB2, PBE1, PUX3, RGLG2, RHA2A, RIN2, ROC1, RPN10, RPT3, RPT5B, SAG12, scpl49, SKP2A, SKP2B, UBC2, UBC28, UBQ3, UBQ9, UCH3, XBCP3*
Proteolyitc enzymes (down)	*ATL3, ATL5, ATRBL2, EGY1, EMB2083, emb2458, FKF1, ftsh9, GRH1, MUB5, PIP, SBT1.3, scpl10,* *scpl2, scpl20, scpl25, scpl42, SKP1B, SLP2, SLP3, SLY2, SNG1, UBC20, UBC29, UBC7, UBP24, V*	*DEGP8, FTSH1, FTSH11, nClpP6, PT, RUB1, UBC8*
Secondary metabolic(up)	*CYP73A5, CYP81F2, FAH1, pal1, PGGT-I, SUR2, UGT72E1*	*4CL5, ALDH10A8, ALDH10A9,ATCPISCA,BCAT4, CYP79F2, DXPS1, ELI3-2, LAS1, MBP1, NIC2, SIAA1, SRG1, SS2, TGG2, TPS04, TPS10, TT3, VTE2*
Secondary metabolic (down)	*ABC4, AOP1.1, AOP2, BCAT3, CYP706A5, FPS2, GGPS1, IPP2, ISPH, KCS5, LAC11, LAC17,* *LUP1, MVA1, PMG1, PMG2, REF2, TT4, YRE,*	*CAC3, CAD4, DXS, FLS, HCT, KCS10, LUT2, PAL3, PDE277, PEN2, POP1, PSY, SPS2, TT5, VTE3*
Cell wall (up)	*AGP5, ATHRGP1, ATPME3, BXL1, CSLE1, EXP16, FUT4, FUT7, GER1, GER2, MUR_1, UXS4,* *XTH22, XTR4*	*ATAGP1, ATAGP10, AtAGP24, CSLA01, CSLG1, DIN9, ISA1, KING1, MEE31, PGAZAT, PGIP2, PMEPCRA, RGP1, UGE3*
Cell wall (down)	*AGP7, AGP9, ATAGP12, ATAGP18, ATAGP19, AtAGP21, ATAGP22, ATAGP26, ATAGP4, ATFUC1, ATFXG1, AtGH9B5, AtGH9B8, AtkdsA1, COB, EXPB1, EXPL2, EXPR, EXT, FLA10, FLA11,* *FLA12, FLA17, FLA18, FLA9, FLR1, LEW2, PMR6, QUA1, UER1, UGE2, UXS3, XTH9*	*ATAGP16, ATAGP25, BGAL2, CSLA03, CSLA7, CSLB03, EXP1, EXP15, LGT1, PRP4, ROL1, SOS5*
TFs (up)	*HSS, HYH, KNAT4, KNAT6S, LBD37, LBD39, LD, LUH, MBD4, MYB15, MYB33, NIMIN-2, NIMIN-3, ORA47, RAP2.4, RAV1, SAI1, SDG15, SIZ1, SNF7.1, SPL, TGA3, TGA5, TOC1, WRKY20, WRKY21, WRKY22, WRKY25, WRKY26, WRKY33, WRKY38, WRKY39, WRKY40, WRKY46, WRKY47, WRKY50, WRKY51, WRKY53, WRKY69, ZAT10, ZAT6, ZAT7, ZCW32, ZFAR1*	*RAP2.6,AGD5, ARR2,ATHB7, AtIDD11, ATMYB102, AtMYB32, ATNAC3, ATRBP45C, CDC5, GL19, IAA18, LCL1, LHW, LOL2, LZF1, MBD11, MYB112, MYB114, MYB59, MYB95, PAP1, PAP2, PHV, PMZ, PRR2, PUR, RAP2.12, RAP2.3, Rap2.6L, RPD3A, TOM1, ZFP7, ZFP8*
TFs(down)	*ARF11, ARF22, ARF8, ARR12, ATH1, ATRR3, BLH6, BZO2H2, CIA2, ETT, GBF5, HDT2, HMGB6,* *IAA16, ICU4, MBD10, MEE47, MS1, MYB124, NGA2, OBP4, PCNA2, pde191, PMG1, PMG2,* *PTAC1 RAP2.2, RR16, SAW2, SDG26, SHY1, STH, TCP4, TINY2, UNE10, VPS46.1, VRN2, WHY3,* *WLIM1, WOX4, FHD2*	*TCP24, anac061, ARR7, ATCTH, ATHB5, BEE2, COL3, EIL1, HAT2, hda14, IAA1, IAA8, IBC6, METI, MFP1, MSG2, MYB20, MYB60, PDF2, PIL5, PIL6, PTAC4, TRY, WRKY11, WRKY30, ZFN1, ZFP4*
Ethylene (up)	*ERF11, ERF5, ERF2, ERF-6-6, ERF13, ORA59, RAP2.5, ERS1, ERF7,atpdx1.2,*	*MBF1B, ERF3*
Ethylene (down)	*2-oxoglutarate-dependent dioxygenase*	*ACS10*
ABA (up)	*STO1, AAO3*	*SIR3, FIP1*
ABA (down)	*ATHVA22C, HVA22D*	*HVA22H*
JA (UP)	*LOX5*	*LOX1, CYP74A, AOC2, AOC4, JMT, JR1*
IAA(UP)	*WIN3, AIR12, AXR1, TIR5, ILL1, ARG1*	*TGG2, GH3-10, WES1, YDK1, ILR1, GH3.6, ILL5*
IAA(Down)	*AFB2, COV1, MES17*	*SAUR_AC1*
SA (UP)	–	BSMT1, UDP-glucoronosyl
SA(Down)	methyltransferase	

* Only those genes with an alias (short annotation name present in TAIR) have been included in this summary table. A complete list of aphid-specific and *P. syringae*-specific genes and their corresponding At IDs have been provided in **Table S6.**

This study therefore demonstrates that the integration of heterogeneous publicly available information from multiple databases with experimental results can help plant biologists develop a better understanding of stress-associated processes in plants. Due to logistics and costs we examined only a single time point during the *A. thaliana* (Col-0) - *P. syringae* treatment. We are fully aware that comparing single time point restricts some analyses and is a potential limiting factor as demonstrated by Bricchi et al (2012) [62]. Although several datasets reporting temporal responses of *A.thaliana* to *P. syringae* infection were available from previously published independent studies [33], [63], [64], [65], we decided not to combine them in the current analysis while making comparisons with our own *B. brassicae* data [66] to maintain the homogeneity of the comparisons. The analysis presented here will therefore not explain the comparative temporal dynamics of *A. thaliana* – *B. brassicae* and *A. thaliana – P. syringae* interactions.

## Materials and Methods

**Figure 11:**
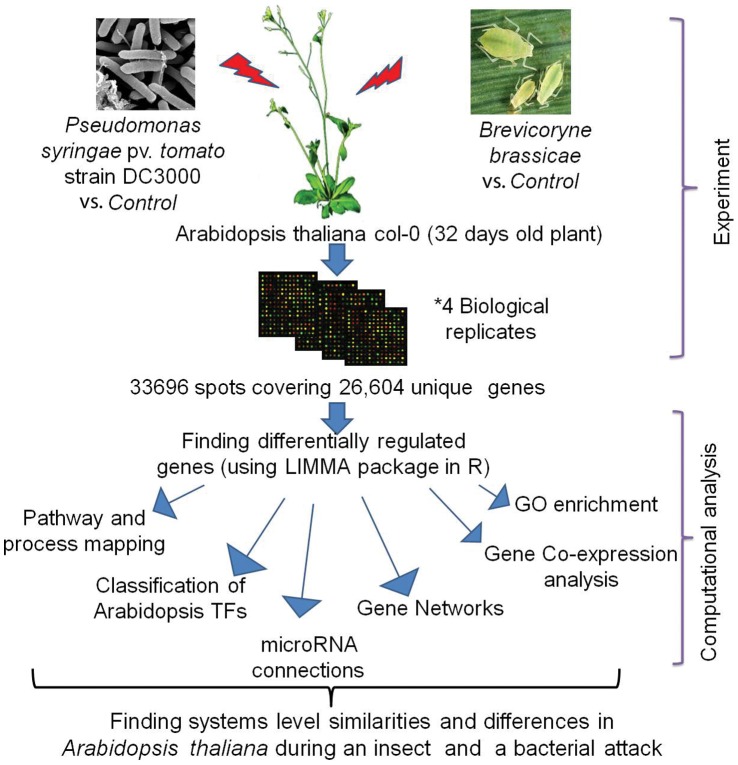
Flow chart of the methodology.

To overcome the problem of the incompatibility of independent microarray experiments, a genome-wide expression analysis involving 2 different biotic stresses was conducted, in which *Arabidopsis thaliana* plants were infested with aphids (*Brevicoryne brassicae*) [45] or infected with *P. syringae* bacteria (4 biological replicates, and an untreated control were used for each comparison). The microarray data from the aphid experiment was part of a larger plant-insect study [45]. The Pseudomonas data were generated for the present study using the same technology platform to reduce experimental variation. All data have been deposited in GEO (**GSE39245** and **GSE39246**). A systems biology approach was followed to understand common and specific responses in terms of different pathways and processes in *Arabidopsis* during insect and bacterial attack. A simplified flow chart diagram of the applied methodology is provided in **Figure 11**.

### Plant Material and Cultivation

The *Arabidopsis thaliana* Columbia-0 ecotype (Col-0) plants used in the experiment were derived from seeds produced by Lehle Seeds (Round Rock, USA; Catalog No. WT-2-8, Seed Lot No. GH195-1). The seeds were sterilised according to standard procedures and grown on agar medium containing an MS basal salt mixture (Sigma), 3% (v/w) sucrose, and 0.7% (v/w) agar (pH 5.7) to assure uniform germination. After 15 days, the seedlings were transferred to 6 cm diameter pots (3 seedlings per pot) filled with a sterile soil mix (1.0 part soil and 0.5 parts horticultural perlite). The plants were kept in Vötsch VB 1514 growth chambers (Vötsch Industrietechnik GmbH, Germany) under the following conditions: 8 h/16 h (light/dark) photoperiod, 22°C/18°C, 40%/70% relative humidity, and 70/0 µmol m^−2^s^−1^ light intensity. A short day length was applied to prevent the plants from bolting.

### Infestation Experiments

At 32 days of age (17 days after being transferred to soil), the plants had 8 fully developed leaves. Each plant was infested with 32 wingless aphids (4 per leaf), which were transferred to the leaves with a fine paintbrush. Infested plants and aphid-free controls were maintained in Plexi-glass cylinders, as described previously [66]. The plants were harvested 72 h after infestation between the 6th and 8th hours of the light photoperiod. Four biological replicates were produced from the control and infested plants, with each being sampled from 15 individual plants. Whole rosettes were cut at the hypocotyl, and aphids were removed by washing with Milli-Q-filtered water. The harvested material was immediately frozen in liquid nitrogen.

A *P. syringae* culture was grown overnight in 10 ml of Kings B solution (King *et al*., 1954) supplemented with the antibiotics rifampicin (50* µ*g ml^−1^) and kanamycin (25* µ*g ml^−1^). The overnight culture was washed once in 10 mm MgCl_2_, and the final cell densities were adjusted to an OD of approximately 0.20 at 600 nm (approximately 1.5×10^8^ cfu ml^−1^) in 10 mm MgCl_2_. Plants were grown as described in the Plant material and cultivation section. Then, 30-dayold plants were mock-challenged with 10 mm MgCl_2_ or inoculated with the DC3000 strain of *P. syringae* by infiltrating 3–4 leaves on the abaxial surface with a needleless 1 ml syringe. Four biological replicates of infested leaves and leaves obtained from control plants grown under identical conditions were harvested after 3 days (between the 6th and 8th hours of the light photoperiod). The leaf material was immediately frozen in liquid nitrogen. Leaves from 15 plants were included in each replicate.

### RNA Isolation, cDNA Synthesis, Labelling and Hybridisation

Total RNA was isolated from cauline leaf tissue from plants from each experiment. Each experiment consisted of four infested samples and four control samples. Total RNA was extracted from 100 mg of cauline leaf material using the RNeasy Plant Minikit (Qiagen, Hilden, Germany) and eluted in 2×50 µl of RNAse-free water. Any residual DNA in the RNA samples was removed by on-column treatment with RNAse-free DNase. The eluted RNA was concentrated to 10–20 µl using a 30 kDa cut-off Microcon spin filter unit (Amicon, Bedford, USA). To protect the RNA from degradation, the RNasin Plus RNase inhibitor (Promega, Madison, USA) was added to a final concentration of 1 unit µl-1. The purity and quantity of the obtained RNA was determined using a Nanodrop ND 1000 instrument (Nanodrop Technologies, Wilmington, DE, USA). RNA integrity was analysed via formaldehyde agarose gel electrophoresis. First-strand cDNA was generated from total RNA (15 µg) using the Superscript III Reverse Transcriptase (Invitrogen, Carlsbad, USA) and oligo dT primers with a 3DNA capture sequence from the 3DNA Array 350TM kit (Genisphere, Hatfield, USA). RNA samples were labelled with either the Cy5-capture primer or Cy3-capture primers (sample dye-swapping). The cDNAs were hybridised to the microarray slides at 58°C using a Slide Booster Hybridisation Station (Advalytix, Brunnthal, Germany) together with Cy3- and Cy5-labelled dendrimers from Genisphere. The slides were washed according to the manufacturers’ instructions (Genisphere and Advalytix).

### Microarrays

The microarray slides contained 31811 unique 70-mer oligos with a C6-amino linker, corresponding to a total of 33696 spots, covering 26624 genes. Of these oligos, 29110 were from the Qiagen-Operon *Arabidopsis* Genome Array Ready Oligo Set (AROS), Version 3.0, while the others were custom made and produced by Operon (Alameda, CA, USA) or MWG (Ebersberg, Germany). The sequences of all of the custom-made probes on the chip have been deposited in GEO and are available under accession **GPL15699**. The oligonucleotides were dissolved in MQ grade water and 50% DMSO (20 pmol/µl) and spotted on aminosilane-coated UltraGaps slides (Corning, NY, USA) using a BioRobotics MicroGrid II robot (Genomic Solutions, MI, USA). Printing of the microarray slides was performed at the Norwegian Microarray Consortium (Trondheim, Norway). Hybridisations were conducted using a Slide Booster Hybridization Station (Advalytix, Brunnthal, Germany), and the slides were washed according to the manufacturer’s instructions (Genisphere and Advalytix). The slides were scanned at a 10 µm resolution on a G2505B Agilent DNA microarray scanner (Agilent Technologies). The resulting images were processed using GenePix 5.1 software (Axon Instruments, Union City, USA).

### Statistical Analysis of the Microarray Data

Each dataset obtained from the aphid and *Pseudomonas* treatments corresponded to 4 microarray slides, where the controls and treated samples were alternately labelled with Cy5 and Cy3. The GenePix-processed data were filtered to remove spots that had been flagged as ‘Absent’, ‘Not Found’ or ‘Bad’, or exhibited median foreground intensity below the local median background intensity. The R statistical program (version 2.10.1) was used for all statistical analyses [67]. No background subtraction was performed. The data from each array were log-transformed and normalised using the printtip-loess approach (Yang et al. 2001). Within-array replicated measurements for the same gene were merged by taking the average over the replicates. The data were then scaled so that all array datasets presented the same median absolute deviation. Genes showing dye-biased responses due to Cy5 and Cy3 labelling were identified and excluded. During data processing, we focused on genes for which at least 3 out of 4 biological replicates for the examined time points passed the quality control criteria suggested by Jørstad et al. [68], [69]. To make statistical inferences about differentially regulated genes, the Limma package [70] was used. The Limma approach is based on fitting a linear model to the expression data from each probe on a microarray. Genes showing an adjusted p-value of less than 0.05 were considered to be significantly differentially expressed. All of the genes discussed in this paper were found to be significantly differentially expressed in one of the two treatments (aphid or *Pseudomonas*).

### GO Enrichment Analysis of Common Genes

We employed a simple set theory-based operation in R to find common and specific transcriptional responses that occurred in both experiments. To conduct automated GO [71], TAIR [72] annotations, we simultaneously used three programs: ClueGO [73], BiNGO [74] and *VirtualPlant* [75]. Only the ClueGO results were included in this manuscript. Transcription factors were classified according to the ‘The Database of Arabidopsis Transcription Factors’ [76]. In ClueGO, to calculate enrichment values for terms and groups, we used two-sided (enrichment/depletion) tests based on the hypergeometric distribution to calculate doubling for two-sided tests to address discreetness and conservatism effects, as suggested by Rivals et al. [77]. To correct the P-values for multiple testing, the Bonferroni method was used to control the type I error (false positive) rate [78]. ClueGO employs a new kappa statistic. To link the terms in the network, ClueGO first creates a binary gene-term matrix with the selected terms and their associated genes. Based on this matrix, a term–term similarity matrix is calculated using chance-corrected kappa statistics to determine the strength of the associations between the terms. Because the term–term matrix is of categorical origin, using a kappa statistic was found to be the most suitable method. Finally, the created network represents the terms as nodes, which are linked based on a predefined kappa score level. The kappa score threshold can initially be adjusted on a positive scale from 0 to 1 to restrict the network connectivity in a customised way. In our analysis, we used a kappaScore threshold of 0.3. The size of the nodes reflects the enrichment significance of the terms. The functional groups represented by their most significant (leading) term are visualised in the network, providing an insightful view of their interrelationships. Furthermore, other ways of selecting the group-leading term, e.g., based on the number or percentage of genes per term, are also provided.

*VirtualPlant* [75] integrates genome-wide data regarding the known and predicted relationships among genes, proteins, and molecules as well as genome-scale experimental measurements. This warehouse includes descriptions of molecular entities (e.g., gene annotations and functional classifications), molecular interactions (metabolic associations, regulatory interactions, and other interaction data from public databases), and publicly available microarray data (including more than 1,800 gene chip hybridisations from the ATH1 Affymetrix platform obtained from the European Arabidopsis Stock Center [NASC] using the Affywatch subscription service). VirtualPlant also provides visualisation techniques that render multivariate information in visual formats that facilitate the extraction of biological concepts.

### Co-expression Analysis of Common Genes using CORNET

The construction of co-expression networks for multiple input genes was conducted using the CORNET tool [31]. The co-expression tool calculates the correlation between gene expression profiles using one or more precompiled expression datasets and, as such, identifies possible functional associations between genes. Out of all of the available expression data, we selected the subgroup consisting of 69 ATH1 AtGenExpress biotic stress compendium expression data. All the expression data were processed using RMA from the R BioConductor package and making use of the CDF described in Casneuf et al. [79]. Pearson’s correlation coefficients were calculated between the given genes. Correlation coefficients higher and lower than a certain value are reported. Pearson’s correlation coefficient (PCC) was used at a cut off ≥0.7. Networks and associated evidence were visualised in Cytoscape 2.7.0.

### Gene Networks, microRNAs and Connections to Post-transcriptional Gene Regulation

The *Gene networks* tool in *VirtualPlant* groups individual genes into a supernode based on shared functional properties, such as GO terms, KEGG pathways, gene families and even similar annotations. Edges were drawn between two supernodes when at least one gene or gene product in each supernode showed a molecular interaction. To improve the regulatory interaction predictions, we filtered the transcription factor:target gene predictions to include only the transcription factor and target pairs whose expression values were correlated in the microarray experiment [80]. The selected statistic for the calculation of correlations in this analysis was the Pearson’s correlation coefficient, with a cut-off value of less than or equal to 0.7. The results were then cross-compared with all of the microRNA genes curated in the microRNA Registry (microrna.sanger.ac.uk/sequences) [81] and in the Arabidopsis Small RNA Project (ASRP) Database [82]. In certain cases, we also compared the results with microRNAs and precursor candidates predicted for the *A. thaliana* genome by the algorithm *findMicroRNA* [83]. We followed specific criteria required for the annotation of plant microRNAs, including experimental and computational data as well as refinements of standard nomenclature, as described in [84] [85].

## Supporting Information

File S1 GO-annotation cytoscape network file (.cys) for common genes.
(CYS)

File S2 GO-annotation cytoscape network file (.cys) for aphid-specific genes.
(CYS)

File S3 GO-annotation cytoscape network file (.cys) for *P. syringae*-specific genes.
(CYS)

File S4 MapMan input file for all aphid-responsive genes and corresponding log2-transformed expression values.
(XLS)

File S5 MapMan input file for all *P. syringae-*responsive genes and corresponding log2-transformed expression values.
(XLS)

File S6 Lists of aphid-specific and *P. syringae*-specific genes and their log2-transformed expression values related to biotic stress signalling processes.
(XLS)

File S7 List of the 31 heat shock protein (HSP) genes that were differentially expressed only during aphid treatment and their log2-transformed expression values.
(XLS)

File S8 List of the proteolyitc enzymes differentially expressed during the aphid and *P. syringae* treatments.
(XLS)

File S9 List of genes related to secondary metabolic processes that were differentially regulated during the aphid and *P. syringae* treatments.
(XLS)

File S10 List of genes involved in cell wall precursor synthesis that were differentially regulated during the aphid and *P. syringae* treatments.
(XLS)

File S11 A comparative list of the differentially expressed genes (aphid-specific and *P. syringae*-specific genes) involved in hormonal pathways with their corresponding log2-transformed expression values.
(XLS)

File S12 Details of all of the TFs that were differentially expressed during the aphid and Pseudmonas treatments.
(XLS)
